# National and regional prevalence of posttraumatic stress disorder in sub-Saharan Africa: A systematic review and meta-analysis

**DOI:** 10.1371/journal.pmed.1003090

**Published:** 2020-05-15

**Authors:** Lauren C. Ng, Anne Stevenson, Sreeja S. Kalapurakkel, Charlotte Hanlon, Soraya Seedat, Boniface Harerimana, Bonginkosi Chiliza, Karestan C. Koenen

**Affiliations:** 1 Department of Psychology, University of California Los Angeles, Los Angeles, California, United States of America; 2 Department of Epidemiology, Harvard T.H. Chan School of Public Health, Boston, Massachusetts, United States of America; 3 Duke University Global Health Institute, Durham, North Carolina, United States of America; 4 Centre for Global Mental Health, Health Service and Population Research, Department Institute of Psychiatry, Psychology and Neuroscience King’s College, London, United Kingdom; 5 Department of Psychiatry, Stellenbosch University, Cape Town, South Africa; 6 Faculty of Health Sciences, Western University, London, Ontario, Canada; 7 College of Medicine and Health Sciences, University of Rwanda, Kigali, Rwanda; 8 Department of Psychiatry, Nelson R. Mandela School of Clinical Medicine, University of KwaZulu-Natal, Durban, South Africa; 9 Department of Epidemiology, Harvard T.H. Chan School of Public Health, Boston, Massachusetts, United States of America; Umeå Centre for Global Health Research, Umeå University, SWEDEN

## Abstract

**Background:**

People living in sub-Saharan Africa (SSA) are disproportionately exposed to trauma and may be at increased risk for posttraumatic stress disorder (PTSD). However, a dearth of population-level representative data from SSA is a barrier to assessing PTSD. This manuscript sought to calculate pooled PTSD prevalence estimates from nationally and regionally representative surveys in SSA.

**Methods and findings:**

The search was conducted in PubMed, Embase, PsycINFO, and PTSDpubs and was last run between October 18, 2019, and November 11, 2019. We included studies that were published in peer-reviewed journals; used probabilistic sampling methods and systematic PTSD assessments; and included ≥ 450 participants who were current residents of an SSA country, at least 50% of whom were aged between 15 and 65 years. The primary outcomes were point prevalence estimates of PTSD across all studies, and then within subgroups. The protocol was registered with the International Prospective Register of Systematic Reviews (PROSPERO) (registration number CRD42016029441). Out of 2,825 unique articles reviewed, 25 studies including a total of 58,887 eligible participants (54% female) in 10 out of the 48 countries in SSA were identified. Most studies enrolled any adult aged 18 years or older. However, some studies only enrolled specific age brackets or persons as young as 15 years old. Six studies were national surveys, and 19 were regional.

There were 4 key findings in the meta-analysis: (1) the overall pooled prevalence of probable PTSD was 22% (95% CI 13%–32%), while the current prevalence—defined as 1 week to 1 month—was 25% (95% CI 16%–36%); (2) prevalence estimates were highly variable, ranging from 0% (95% CI 0%–0%) to 74% (95% CI 72%–76%); (3) conflict-unexposed regions had a pooled prevalence of probable PTSD of 8% (95% CI 3%–15%), while conflict-exposed regions had a pooled prevalence of probable PTSD of 30% (95% CI 21%–40%; *p* < 0.001); and (4) there was no significant difference in the pooled prevalence of PTSD for men and women. The primary limitations of our methodology are our exclusion of the following study types: those published in languages other than English, French, and Portuguese; smaller studies; those that focused on key populations; those that reported only on continuous measures of PTSD symptoms; and unpublished or non–peer-reviewed studies.

**Conclusions:**

In this study, PTSD symptoms consistent with a probable diagnosis were found to be common in SSA, especially in regions exposed to armed conflict. However, these studies only represent data from 10 of the 48 SSA countries, and only 6 studies provided national-level data. Given the enormous heterogeneity expected across the continent, and also within countries and regions, this review cannot speak to rates of PTSD in any regions not included in this review. Thus, substantial gaps in our knowledge of PTSD prevalence in SSA remain. More research on population-level prevalence is needed to determine the burden of trauma symptoms and PTSD in SSA and to identify acceptable and feasible approaches to address this burden given limited mental healthcare resources.

## Abstract 2

Lauren Ng and co-workers investigate the prevalence of post-traumatic stress disorder in countries in sub-Saharan Africa.

## Author summary

**Why was this study done?:**

- Repeated and prolonged exposure to violence, armed conflict, and mass-casualty events, combined with a lack of access to mental health treatment, may result in a substantial effect on the population burden of posttraumatic stress disorder (PTSD) in sub-Saharan Africa (SSA).
- While many studies of PTSD have been conducted in SSA, most of these studies derived their estimates from nonrepresentative samples or specific populations.
- Population-representative epidemiologic data are critical to understand the burden of PTSD in SSA and develop national and regional policies to address that burden.

**What did the researchers do and find?:**

- We conducted a systematic review and meta-analysis of the prevalence of PTSD from representative national and regional studies in SSA.
- Pooled prevalence estimates were calculated across all studies, and then within subgroups including by sex, assessment time frame (i.e., 1 week, 1 month, 1 year), use of a screening or diagnostic measure, and whether populations were affected or not affected by mass-casualty war or armed conflict.
- We identified 25 unique studies (*N* = 58,887) across 10 of the 48 SSA countries.
- The pooled prevalence of probable PTSD across all studies was 22% (95% CI 13%–32%).
- The pooled prevalence of probable PTSD in participants from war-exposed regions was 30% (95% CI 20%–40%), while the estimate from war-unexposed regions was 8% (95% CI 3%–15%; *p* = 0.01).

**What do these findings mean?:**

- These data suggest that PTSD symptoms and probable PTSD are common in SSA.
- However, information was only found on 10 of the 48 SSA countries, and only 6 studies provided national-level data.
- Only one study used a measure of PTSD symptoms whose reliability and validity had been assessed previously in the population of interest.
- Our results suggest both that PTSD is a major public health problem in SSA and that large gaps in our knowledge of this problem remain.

## Introduction

Mental and substance use disorders account for 23% of years lost to disability, making them the leading cause of disability worldwide [1]. Posttraumatic stress disorder (PTSD) is a large contributor to the global burden of disease and is estimated to affect almost 4% of the world’s population [2]. PTSD persists for over a year in 50% of all cases [2] and often leads to substantial declines in functioning and productivity [3]. National and regional data on prevalence are used to develop policies and action plans for addressing PTSD and other related disorders. Recently, much of the data on global and national estimates of PTSD has come from the World Health Organization’s World Mental Health (WMH) surveys of the cross-national prevalence of PTSD in 26 countries [2,4,5]. The WMH surveys collected representative population data across the world using structured diagnostic measures to assess PTSD, which allowed for the calculation of global population prevalence estimates of PTSD [2]. However, the WMH surveys only included one national survey and one regional survey from sub-Saharan Africa (SSA); in addition, the one national estimate was from South Africa, one of the few upper-middle–income countries in SSA [2]. The limited number of countries from SSA contributing data on PTSD in the WMH surveys is consistent with the lack of population mental health data from SSA generally [6,7].

People living in SSA may be disproportionately affected by individual and population-level trauma exposure. Indeed, research from the World Health Organization has found that the lifetime prevalence of road traffic deaths [8] and of reported intimate partner violence and/or nonpartner sexual violence are highest in the Africa region [9,10]. In addition, although other regions of the world experience more natural disasters, the great majority of countries most vulnerable to natural disasters are in SSA [11]. SSA has also been disproportionately affected by war and armed conflicts, many of which have been ongoing for years, if not decades [12]. In 2019, 20 countries in SSA were classified by the World Bank as hosting fragile and conflict-affected situations, which represents more than 50% of the fragile and conflict-affected countries globally [13]. Moreover, the legacy of violence, loss, and historical trauma inflicted on the people in SSA through colonization may contribute to high rates of posttraumatic stress [14].

Increased exposure to traumatic life experiences in SSA is compounded by very low rates of access to mental health treatment [15,16]. Of the 48 countries in SSA [17], 24 (50%) are low-income countries, 18 (37.5%) are lower-middle–income countries, and the remaining 6 (12.5%) are upper-middle–income countries [18]. It is estimated that the gap between those who need mental healthcare and those who receive it often exceeds 90% in low-income countries for most mental disorders [19–21]. It is estimated that 77% of people with PTSD in lower-middle–income countries have not received treatment [2]. Given that the majority of countries in Africa are low-income or lower-middle–income, it is likely that the vast majority of people with PTSD in SSA will never receive treatment and are at high risk for chronic symptoms. Repeated and prolonged exposure to violence, armed conflict, and mass-casualty events, combined with a lack of access to mental health treatment [15,16], may result in a substantially larger effect on the population burden of PTSD in SSA [22,23].

### Aims

The goal of this meta-analysis was to synthesize the existing data on the population prevalence of PTSD in SSA. While many studies of PTSD have been conducted in SSA, most of these studies derived their estimates from nonrepresentative samples or specific populations such as refugees or internally displaced persons (IDPs) [24–30], patients [31–35], parents [36–40], or students [41–49]. While studies have summarized PTSD prevalence in conflict-affected populations, including those in SSA [50,51], PTSD occurs in response to a wide range of traumas (both interpersonal and non-interpersonal) that occur in non-conflict settings (e.g., car accidents, sexual assault). Population-representative epidemiologic data are critical to understand the burden of PTSD in SSA and develop national and regional policies to address that burden. To our knowledge, this is the first meta-analysis to summarize the data on population-based point prevalence of PTSD in SSA across all settings. The objectives of this paper were to (a) conduct a systematic review and meta-analysis of the prevalence of PTSD from representative national or regional studies and (b) explore the association between sex, population-level exposure to armed conflict, and pooled prevalence of PTSD.

## Methods

### Search strategy and selection criteria

Study inclusion criteria are as follows:

1. Participants were current residents of a country in SSA (e.g., citizens, permanent residents, IDPs, or refugees or immigrants who had resided in the country for at least 6 months).
2. In order to capture studies that focused primarily on adults, at least 50% of participants had to be between 15 and 65 years of age, which is defined as the “working age population” by the World Bank [52].
3. PTSD data were reported for at least 450 participants. We selected a priori a sample size cutoff to ensure that we would have enough power to detect prevalence estimates lower than 3.9%, which is the mean lifetime PTSD prevalence across the WMH surveys [2]. A minimum sample size requirement of 450 would allow us to identify prevalence as low as 3.3% with a precision of 1.65% within each study or region [53].
4. Studies used a systematic method of classifying participants as those with or without PTSD. Studies could be included if they used structured or semi-structured interviews—either administered by trained lay interviewers or clinicians—to provide a diagnosis or if they used a symptom checklist or diagnostic assessment and applied a cut-point to classify people as having PTSD or not having PTSD. In addition, we included studies that assessed cultural idioms of distress that were clearly related to symptoms developed in response to a stressful or traumatic event [54]. These are referred to as “post trauma reaction syndromes.” For the purposes of this paper, any participants who were either diagnosed with PTSD following an interview or who scored above a cut-point criterion set by the study authors were considered to have symptoms consistent with a diagnosis of PTSD.
5. Studies employed probabilistic procedures to obtain nationally or regionally representative samples. Representative samples that were limited to a specific sex were also included.
6. Articles were written in English, French, or Portuguese, which covers most of the scientific literature from SSA.
7. Studies were published in a peer-reviewed journal with no journal date restrictions.
8. If studies included a mixture of eligible and ineligible participants and data from the group meeting inclusion criteria could be disaggregated, the data that met inclusion criteria were included in the review.

The search strategy was developed and initially conducted in PubMed between February 22, 2016, and August 1, 2017. The search was expanded to 4 databases and rerun using PubMed, Embase, PsycINFO, and PTSDpubs between October 18, 2019, and November 11, 2019. Search terms were “[Name of SSA Country] AND PTSD (in any field).” The search was run for each country in SSA. Using Kenya as an example, PubMed automatically generates the following search when these terms are entered: (“Kenya” [medical subject heading (MeSH) Terms] OR “Kenya” [All Fields]) AND (“stress disorders, post-traumatic” [MeSH Terms] OR (“stress” [All Fields] AND “disorders” [All Fields] AND “post-traumatic” [All Fields]) OR “post-traumatic stress disorders” [All Fields] OR “ptsd” [All Fields]). The full texts of articles that appeared to meet inclusion criteria based on the abstract were downloaded, and references were reviewed to identify additional papers that might meet the eligibility criteria.

**Fig 1:**
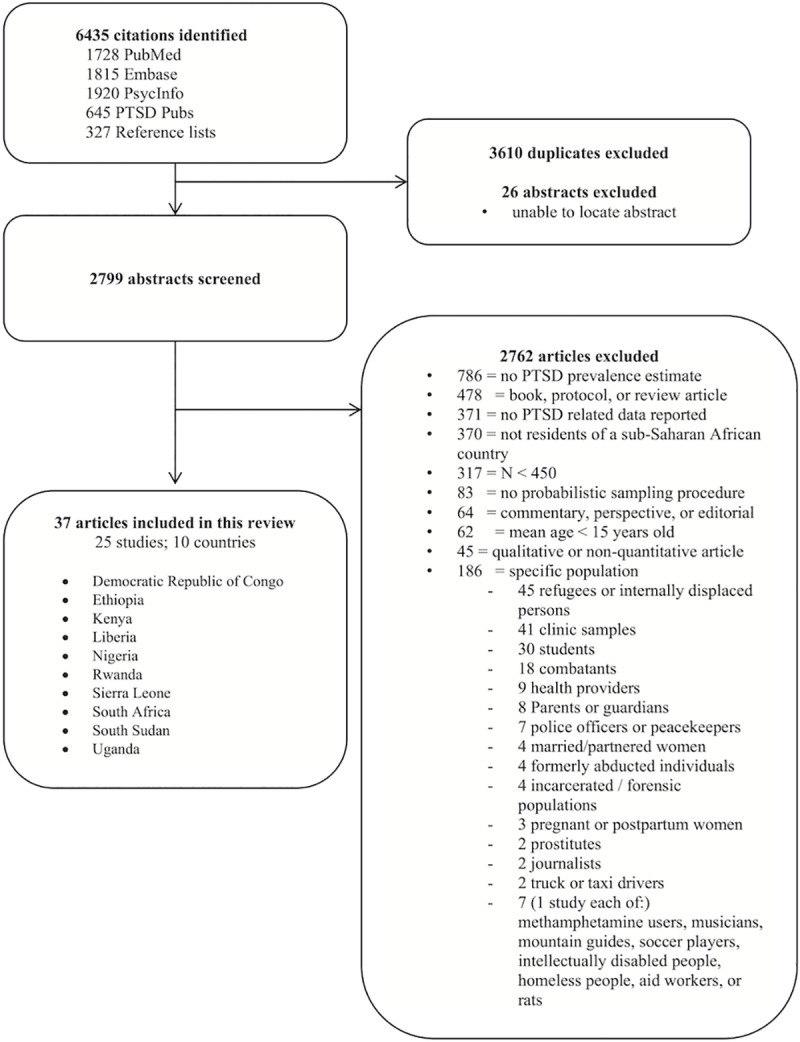
Study selection flow diagram. PTSD, posttraumatic stress disorder.

The Preferred Reporting Items for Systematic Reviews and Meta-Analyses (PRISMA) [55] (see Fig 1 for complete PRISMA flow diagram) process was followed to identify and include/exclude the papers in this review. Citations, abstracts, and full-text articles (when available) for all potentially eligible articles were downloaded and double coded by a team of 8 researchers to ensure they met criteria.

Articles were identified through the PubMed, Embase, PsycINFO, and PTSDpubs search and by reviewing references from the identified papers. Seven fields were then extracted from each paper and entered into a Google Docs spreadsheet: article title, year, first author, journal, search date, researcher conducting the search, and abstract. Duplicate entries were flagged and filtered out of the spreadsheet. To identify eligible studies from the remaining articles, the full text of each article was reviewed, and one coder entered information related to each inclusion and exclusion criteria into the database. A second coder independently reviewed each of the inclusion and exclusion criteria to assess concordance. Disagreements about study eligibility were reviewed and discussed by the research team, and final decisions were made by consensus.

The abstracts of non-English articles were reviewed by native speakers of the article language to determine eligibility, and if the abstract was deemed eligible, the full article was reviewed by the native speaker and also underwent targeted translation into English to enable accurate data extractions by a second coder. Studies that were not related to the eligibility criteria (e.g., a commentary or a study describing physical trauma instead of psychological trauma) were flagged and removed. For studies for which it was unclear whether they met eligibility criteria, the first author (L.C.N.) emailed the authors of the study to clarify.

Study quality was assessed using a standardized tool for observational studies [56]. Loney and colleagues (1998) was selected against a range of checklists and scales based on its applicability to prevalence studies, validation, reliability, and clear methodology for rating studies [57]. The tool contains 8 items, each with a possible score of 1, with a maximum total score of 8.

In implementing the quality assessment tool, we operationalized 4 of the questions on the Loney tool to best suit the purposes of this systematic review and meta-analysis:

1. On the first question (“Are the study design and sampling method appropriate for the research question?”), a full point was given if the study was a whole population or a random sample and the authors described the process in enough detail to support the statement from region down to the randomization of the individual selected to participate. Studies received a 0 if they were not the whole population or a random sample, or a half point if the study design was described as a “random sample”, but there was not enough evidence to confirm this.
2. On the fourth question (“Are objective, suitable, and standard criteria used for measurement of the health outcome?”), we defined this as “PTSD measurement tools which are consistent with accepted clinical/research criteria and validated in the population of interest.” If the battery was an acceptable clinical/research tool but had not been validated during the study or previously in the population of interest, it received a half point.
3. On the fifth question (“Is the health outcome measured in an unbiased fashion?”), we adapted this to make it more appropriate to our study framework. The original tool recommends blinding interviewers to the purpose of the study in some cases. We did not feel this was applicable in the context of prevalence studies of PTSD in SSA as most teams employed interviewers to conduct the batteries and trained them specifically on research methods in order to implement the study. Papers received a full point on this question if they described the assessors and described the training that they received to conduct the interviews and if internal reliability was calculated. If only one of these items was stated in the paper, the question was scored as a half point.
4. On the sixth question (“Is the response rate adequate? Are the refusers described?”), papers received a full point if the response rate was at least 70% and if the authors described any demographic details about the refusers. Studies scored a 0 if they did not meet the response rate threshold and did not describe the refusers, or a half point if they did one or the other. All other items on the Loney tool were scored as either 0 or 1 with no half points. The association between study quality and prevalence estimate was assessed using a meta-regression analysis.

This systematic review was registered in the International Prospective Register of Systematic Reviews (PROSPERO) on December 1, 2016, and updated on June 21, 2019 (registration number CRD42016029441; https://www.crd.york.ac.uk/prospero/display_record.php?RecordID=29441&VersionID=52136).

### Data analysis

Data were extracted from eligible articles by author L.C.N., and all extractions were reviewed and confirmed by at least one other researcher. The primary outcomes of interest were the point prevalence estimates of PTSD. In addition, information on the number, age, and sex of participants; population-level trauma exposure; sampling procedures; and the language, translation, validity, and reliability of the PTSD assessment tools were also extracted.

**Fig 2:**
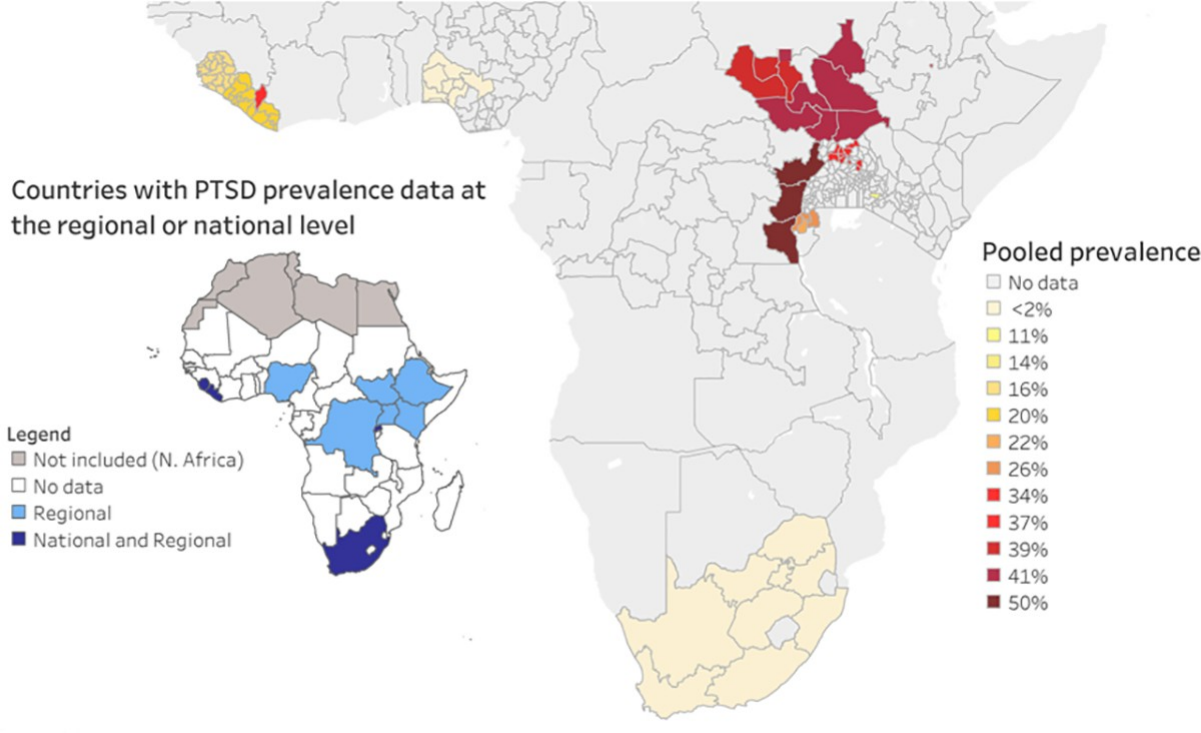
PTSD in SSA: Pooled prevalence from countries with regionally or nationally representative data. PTSD, posttraumatic stress disorder; SSA, sub-Saharan Africa.

Pooled prevalence estimates were calculated across all studies, and then within subgroups including by sex, assessment time frame (i.e., 1 week, 1 month, 1 year), use of a screening or diagnostic measure, and whether populations were affected or not affected by mass-casualty war or armed conflict. War or armed conflict was defined using the international humanitarian law definition, in which armed conflict occurs between organized armed groups, governmental or nongovernmental [58]. Pooled prevalences at the national and regional level were calculated to produce a heat map of available PTSD estimates in SSA (see Fig 2). Pooled estimates were calculated using the Stata version 14.2 [59] metaprop [60] command, which allows for the inclusion of all studies, including those with 0% or 100% prevalence proportions. Metaprop was run using (a) a random-effects model, which assumes that differences in prevalence estimates are not solely due to sampling error, (b) the exact confidence intervals, and (c) the Freeman-Tukey double arcsine transformation to normalize the prevalence estimates prior to pooling [60]. Q and *I*^2^ were calculated to assess heterogeneity across all studies and within and between subgroups [61]. Finally, a meta-regression with all a priori–defined subgroups in which significant differences in pooled prevalence were identified was run using the Stata metareg command [62].

Weighted prevalence estimates were used if they were reported; otherwise, raw numbers of participants with PTSD and total sample size were included as data. If raw numbers of participants with PTSD were not reported, they were calculated from summary data reported in the manuscripts. Prevalence estimates were calculated based on the total number of participants included in the studies regardless of whether or not they reported experiencing a traumatic event. In some cases, publications only reported conditional PTSD prevalence estimates (i.e., PTSD prevalence only among people who reported a traumatic event). In cases in which the manuscript only reported conditional prevalence, but the overall prevalence estimate could be calculated based on the other information provided in the manuscript, the overall prevalence estimate was calculated and was included in the meta-analysis. Study authors were contacted for clarification by L.C.N. in cases for which prevalence estimates were ambiguous.

## Results

A total of 6,435 articles were identified through database and reference searches, of which 2,825 were unique articles (see Fig 1 for complete PRISMA flow diagram). After removing the duplicates and 26 abstracts that could not be located, 2,799 articles remained for full review. The coders disagreed on the eligibility of 4 articles: for 2 studies, the mean PTSD symptom scores were reported but not estimates of diagnostic prevalence [63,64], and 2 studies met all inclusion criteria except that they did not report PTSD scores [65,66]. After review by the first author and in discussion with the coders, consensus was reached on eligibility. Ultimately, 2,762 articles were excluded, leaving 37 that met eligibility. The 37 articles described 25 unique studies across 10 of the 48 SSA countries (see Table 1 and Fig 2).

**Table 1:** Identified studies.

Country	Studies	Population-level trauma exposure	National or regional	Regional, specified	If regional, how/why region was selected	Year of data collection	Inclusion criteria	Response rate	*N* with PTSD data	PTSD measure and scoring	Time frame	PTSD prevalence (95% CI)	Women with PTSD data		PTSD %; sex difference	Age: M (years) (SD/CI); range
**DRC**	**Johnson et al., 2010 [124]**	Violence and armed conflict for decades	Regional	Subregions of the North and South Kivu provinces and the Ituri district	High levels of violence during the war in Rwanda that crossed over the border into the DRC	Mar 2010	Adults (18+)	98.9%	989	PSS-I in Kiswahili; DSM-IV criteria Lay administered	1 month	50.1% (43.8%–56.3%)	593	60.0%	Men: 44.4% (35.4%–53.4%) Women: 54.0% (46.6%–61.5%). *p* = 0.08	Mean = 40.1 (95% CI 38.2–42.0 years)
**Ethiopia**	**Asnakew et al., 2019 [83]**	Deadly garbage landfill landslide	Regional	Area of the Koshe landslide, Addis Ababa, Ethiopia, 2018	Area experienced trauma due to landslide	May and June 2018	Participants (15+ years old)	98.2%	830	PCL-C in Amharic	1 month	37.3% (34.1% to 40.8%)	491	59.2%	Men: 31.3% (26.4%–36.5%) Women: 41.5% (37.1%–46.0%) *p* = 0.003	Mean = 33 years (SD = 12)
**Kenya**	**Jenkins et al., 2015 [125]**	Election violence in August 2007 and 2013	Regional	The Maseno area in Kisumu county, Nyanza Province in western Kenya	Endemic for malaria. In addition, Nyanza province experienced serious election violence in August 2007	6 months in 2013	None reported	96.4%	1,146	TSQ in English, Kiswahili, and Dholuo Cutoff of 6 Restricted to those who experienced trauma after age 16	1 week	10.6% (8.8%–12.5%)	545	47.6%	Men: 6.7% (4.8%–9.0%) Women: 14.9% (12.0%–18.1%) *p* < 0.001	<30 years = 281; 30–60 years = 448; >60 years = 171
**Liberia**	**Galea et al., 2010 [84]**	Armed violence and conflict from 1989 to 2003	Regional	Rural areas of Nimba County	History of civil conflict in Nimba County	Nov and Dec 2008	Analyses restricted to those aged 19+	98.0%	1,376	HTQ in Liberian English Self-reported	1 week	48.3% (45.7%–50.9%)	631	45.9%	Not reported	<35 y = 574 (41.7%); >35 = 802 (58.3%)
	**Johnson et al., 2008 [67]**		National	N/A	N/A	May 2008	Adults (18+), give accurate information about household	98.2%	1,661	PSS-I in Liberian English; DSM-IV criteria Lay administered	1 month	44.0% (38.0%–49.0%)	876	52.7%	Men: 46% (38%–54%) Women: 42% (38%–46%) *p* = 0.17	Mean = 41 (95% CI 40–42 years)
	**Vinck et al., 2013 [68]**		National	N/A	N/A	Nov and Dec 2010	Adults (18+)	93.4%	4,496	PCL-C (language not reported) Cutoff of 44 Information on PCL-C assessors is not provided	1 month	12.6% (11.5%–13.9%)	2,272	50.5%	Men: 6.3% (5.3%–7.4%) Women: 18.8% (17.2%–20.5%) *p* < 0.0001	Mean = 37.4 (SE = 0.26)
**Nigeria**	**Gureje et al., 2006 [98]**	None reported	Regional	Yoruba-speaking areas: Lagos, Ogun, Osun, Oyo, Ondo, Ekiti, Kogi, and Kwara	Not reported	Feb and Nov 2002	Adults (18+), fluent in Yoruba	79.9%	4,984	WMH Survey version of the CIDI in Yoruba Lay administered	12 months	0.0% (0.0%–0.0%)	2,552	51.2%	Men: 0% (0%–0.2%) Women: 0% (0%–0.1%) *p* > 0.95	Mean = 35 (SE = 0.37)
**Rwanda**	**Fodor et al., 2015 [96]**	1994 genocide that followed years of ethnic tensions and conflict	Regional	Ngoma commune in the Huye district in the South Province	Heavily affected by the genocide	Feb and Mar 2011	Adults (18+)	96%	500	PCL-C in Kinyarwanda Cutoff score of 44 Administered by trained native Rwandan college graduates in interview format	1 month	21.6% (18.1%–25.5%)	349	75.0%	Not reported	Mean = 41.06 (14.9); 18–83
	**Munyandamutsa et al., 2012 [70], Eytan et al., 2015 [69]**		National	N/A	N/A	3 months in 2008	Ages 16+, Rwandan, fluent in Kinyarwanda, no mental impairment	Not reported	962	PTSD section of the MINI DSM-IV and ICD-10 criteria Clinician-administered	1 month	26.1% (23.2%–28.9%)	567	58.9%	Men: 20.5% (16.6%–24.8%) Women: 30.0% (26.2%–33.9%) *p* = 0.001	Median = 33; range: 16–108 years
	**Pham et al., 2004 [126]**		Regional	4 communes: Ngoma (known as Butare town), Mabanza, Buyoga, and Mutura)	Diversity in region, level of urbanization, experience with the genocide, and relationship to the ICTR	Feb 2002	Adults (18+)	99%	2,091	PCL-C (language not reported); cutoff of 44 Self-reported	1 month	24.8% (23.0%–26.6%)	1,074	51.4%	Men: 19.6% (17.2%–22.2%) Women: 29.7% (27.0%–32.5%) *p* < 0.001	Mean = 36.4 years; range 18–94 years
	**Rugema et al., 2015 [80]**		Regional	Southern Province	Not reported	Dec 2011 to Jan 2012	Aged 20–35 years, Rwandan	99.8%	913	MINI in Kinyarwanda Clinician-administered	1 month	13.6% (11.4%–16.0%)	477	52.0%	Men: 7.1% (4.8%–9.9%) Women: 19.5% (16.0%–23.3%) *p* < 0.001	20–24 = 275 (30.3%); 25–29 = 300 (33.0%); 30–35 = 333 (36.7%)
**Sierra Leone**	**Betancourt et al., 2016 [97]**	Civil war from 1991–2002 and EVD	Regional	Western area rural district and western area urban district	Diversity in ethnic composition and degrees of war exposure; was epicenter of EVD cases during the 2014–2015 outbreak	Jan to Apr 2015	Adults (18+)	98%	1,008	PSS-I adapted for use in Liberia in Krio Lay (trained research assistants) administered	1 month	6% (4%–7%)	505	50.8%	Not reported	Mean = 34.2 years (95% CI 33.2–35.2)
	**Jalloh et al., 2018 [71]**	Ebola	National	N/A	N/A	Jul 2015	Head of household and another individual (aged between 15 years and 24 years) or a woman	98%	3,564	IES-6 Administered by trained data collectors	1 week	16% (14.7%–17.1%)	1,774	50%	Not reported	Median = 35 (SD = 15)
**South Africa**	**Herman et al., 2009 [75]; Williams et al., 2008 [127]; Atwoli et al., 2013 [72]; Atwoli et al., 2017 [73]; Koenen et al., 2017 [2]; Stein et al., 2008 [74]; Myer et al., 2009 [128]; Duckers et al., 2018 [129]**	Apartheid until 1994. In the post-Apartheid era, high rates of nonpolitical violence and crime, child abuse, IPV, and HIV/AIDS and TB	National	N/A	N/A	Jan 2002 to June 2004	Adult South Africans residing in households and hostels	87.1%	4,351	CIDI version 3.0 in English, Afrikaans, Zulu, Xhosa, North Sotho, South Sotho, and Tswana; DSM-IV PTSD Lay administered	12 months	0.6% (0.4%–0.8%)	2,619	60.2%	Men: 0.6% (0.3%–1.1%) Women: 0.6% (0.3%–1.0%) *p* = 0.92	Not reported
	**Machisa et al., 2017 [85]**		Regional	Gauteng Province	Not reported	2010	Adult women	79%	501	HTQ in English, Zulu, Sotho, and Afrikaans Researcher administered Mean score ≥ 2.5	1 week	11.6% (9%–14%)	495	100.0%	Only women included	18–29 = 30%; 30–44 = 36%; >45 = 33%
	**Peltzer and Pengpid, 2019 [77]**		National	N/A	N/A	2012	Aged 15+	92.6%	15,201	17-item DTS Information on assessors is not provided	1 week	2.1% (1.9%–2.3%)	8,254	54.3%	Men: 2.0% (1.7%–2.4%) Women: 2.3% (2.0%–2.6%) *p* = 0.20	Mean = 36.8 (SD = 16.5)
	**Smit et al., 2006 [82]**		Regional	Periurban settlement outside Cape Town, South Africa	High HIV prevalence	Not reported	Aged 15+	64.3%	645	HTQ in Xhosa and English; a cutoff score of 75 Self-reported	1 week	14.9% (12.2%–17.9%)	357	55.3%	Men: 18.7% (14.0%–24.0%) Women: 13.7% (10.3%–17.7%) *p* = 0.10	Mean = 30.3 (11.9)
	**Topper et al., 2015 [81]**		Regional	Eastern Cape Province, urban and semiurban areas of Nelson Mandela Bay and the semirural area of Kirkwood	Not reported	Not reported	Aged 18–40 years	97.7%	977	MINI, version 6.0.0 in Xhosa, English, and Afrikaans; information on PCL-C assessors is not provided	1 month	10.8% (9.0%–13.0%)	467	47.8%	Men: 11.6% (8.9%–14.7%) Women: 10.1% (7.5%–13.2%) *p* = 0.45	Not reported
**South Sudan**	**Ayazi et al., 2012 [99]; Ayazi et al., 2014 [130]**	Decades of ongoing civil war	Regional	The Greater Bahr el Ghazal region	Not reported	2010	Adults (18+)	95%	1,200	HTQ with minor adaptations; Arabic with key terms in indigenous languages Cutoff of 2.0 Information on HTQ assessors is not provided	2 weeks	37.6% (34.8%–40.4%)	506	44.0%	Men: 33.6% (30.0%–37.4%) Women: 42.1% (37.8%–46.5%) *p* = 0.003	18–25 = 308 (25.7%); 26–35 = 391 (32.6%); 36–50 = 395 (32.9%); >50 = 89 (7.4%)
	**Ng et al., 2017 [131]**		Regional	11 sites in 6 of South Sudan’s 10 states: (Central Equatoria, Jonglei, Upper Nile, Western Equatoria, Eastern Equatoria, and Lakes) and Abyei	Diverse in terms of ethnicity, socioeconomic status, livelihood, exposure to conflict, and security access	Dec 2014 to Apr 2015	Adults (18+); South Sudanese	99.5%	1,520	The HTQ-R in Classical Arabic, Juba Arabic, Dinka, Nuer, Shilluk, and Bari DSM-IV criteria Information on HTQ-R assessors is not provided	1 week	40.7% (38.2%–43.2%)	773	50.9%	Men: 45.2% (41.6%–48.9%) Women: 36.2% (32.8%–39.7%) *p* < 0.001	Mean = 36.93 years (SD 13.90); range 18–86
	**Roberts et al., 2009 [132]**		Regional	Juba Town	High numbers of IDPs, returned IDPs and refugees	20–30 Nov 2007	Adults (18+)	96.2%	1,242	Adapted version of the HTQ in Juba Arabic and Bari Mean PTSD scores ≥ 2.0 Trained lay administered	1 week	36.2% (33.2%–39.4%)	630	50.7%	Men: 29.7% (25.4%–34.5%) Women: 42.5% (39.4%–45.7%) *p* < 0.01	Mean = 33 years
**Uganda**	**Ertl et al., 2014 [79]; Neuner et al., 2012 [78]**	Twenty-one years of war	Regional	Anaka, Awer, and Padibe Acholi regions	Varying degrees of war exposure and distance from the largest town, Gulu	Jul 2007 to Apr 2008	Aged 12–25	99.9%	1,113	PDS in Luo Information on PDS assessors is not provided	1 month	15% (13%–17%)	693	62.3%	Not reported	Not reported
	**Mugisha et al., 2015 [133]; Mugisha et al., 2015 [134]; Mugisha et al., 2016 [135]**		Regional	Subcounties Lalogi and Koro (in Gulu district); Amuru and Atiak (in Amuru district); and Koch Goma and Alero (in Nwoya district)	Some of the most affected subdistricts by the 20-year civil war in northern Uganda that had a health center	2 Jan 2013 to 2 June 2013	Adults (18+), stayed in the area for more than 6 months, could fluently speak Luo	100%	2,361	MINI in Luo Administered by trained psychiatric nurses	1 month	11.8% (10.5%–13.1%)	1,475	62.5%	Men: 13.4% (11.2%–15.7%) Women: 10.9% (9.3%–12.5%) *p* = 0.002	18–24 = 555 (23.5%); 25–34 = 644 (27.3%); 35–44 = 490 (20.8%); 45–54 = 672 (28.5%)
	**Pham et al., 2009 [136]**		Regional	8 districts: Amuru, Gulu, Kitgum, Pader, Lira, Oyam, Amuria, and Soroti	Represent a variety of ethnic groups (Acholi, Iteso, and Langi) and exposure to the armed conflict	Mar to June 2007	Adults (18+)	64.5%	2,867	PCL-C in 3 local languages Cutoff score of 44 Non-clinician administered	1 month	54% (52%–56%)	1,417	49.5%	Men: 40% (37.5%–42.6%) Women: 68.3% (65.8%–70.7%) *p* < 0.001	Mean = 35.4 years (SD = 14.35)
	**Vinck et al., 2007 [137]**		Regional	Four districts: Gulu, Kitgum, Lira, and Soroti	Selected to represent a diversity of ethnic composition (Acholi, Langi, and Teso) and varying degrees of exposure to the war	Apr to May 2005	Adults (18+)	73%	2,389	PCL-C in Acholi, Lango, and Ateso Cutoff score of 44 Information on PCL-C assessors is not provided	1 month	74.3% (72.5%–76.0%)	1,198	50.1%	Men: 64.7% (61.9%–67.4%) Women: 83.3% (81.6%–85.8%) *p* < 0.001	Mean = 37 years (SD = 13.8 years)

**Abbreviations:** CI, confidence interval; CIDI, Composite International Diagnostic Interview [94]; DRC, Democratic Republic of Congo; DSM-IV, Diagnostic and Statistical Manual Version 4 [138]; DTS, Davidson Trauma Scale [89]; EVD, Ebola virus disease; HTQ, Harvard Trauma Questionnaire [86]; HTQ-R, Harvard Trauma Questionnaire-Revised; ICD-10, International Classification of Diseases, Tenth Revision [139]; ICTR, United Nations’ International Criminal Tribunal for Rwanda; IDP, internally displaced person; IES-6, Impact of Event Scale-6 [91]; IPV, intimate partner violence; MINI, Mini International Neuropsychiatric Interview [92]; N/A, Not applicable; PCL-C, PTSD Checklist-Civilian Version [87]; PDS, Posttraumatic Stress Diagnostic Scale [90]; PSS-I, PTSD Symptom Scale Interview [93]; PTSD, posttraumatic stress disorder; SE, standard error; TB, tuberculosis; TSQ, Trauma Screening Questionnaire [88]

Six of the studies were national surveys, including 2 from Liberia [67,68], 1 from Rwanda [69,70], 1 from Sierra Leone [71], and 2 from South Africa [2,72–77]. The other 19 studies were representative samples of a region within a country. Across the 25 studies, data were available from 58,887 participants, 54% of which were women (*n* = 31,672 of 58,795 participants with data on sex). Some studies did not include the raw number of participants by sex, and so the number of women was calculated based on the information available in the paper, such as the percentage of participants who were women. Most studies enrolled any adult (men and women aged 18 years or older). However, some studies only enrolled specific age brackets (e.g., any person aged 12 to 25 [78,79], 20 to 35 [80], and 18 to 40 years) [81]. In addition, 3 studies enrolled any person aged 15 or older [82,83], 1 study enrolled any person 16 and older [69,70], and 1 study enrolled any adult aged 18 or older but restricted analyses to those aged 19 and older [84]. In addition, 1 study only enrolled adult women [85]. There were no studies that only enrolled adult men.

Across the 25 studies, presence of PTSD was assessed using translated, and in some cases adapted, scales (see Table 1). Sixteen studies used self-reported screening instruments administered by trained lay researchers/data collectors, including the Harvard Trauma Questionnaire (HTQ) [86] (6 studies), PTSD Checklist-Civilian Version (PCL-C) [87] (6 studies), Trauma Screening Questionnaire (TSQ) [88] (1 study), the Davidson Trauma Scale (DTS) [89] (1 study), the Posttraumatic Stress Diagnostic Scale (PDS) [90] (1 study), and the Impact of Event Scale-6 (IES-6) [91] (1 study). The other 9 studies used structured or semi-structured interviews, including the Mini International Neuropsychiatric Interview (MINI) [92] (4 studies), the PTSD Symptom Scale Interview (PSS-I) [93] (3 studies), and the Composite International Diagnostic Interview (CIDI) [94] (2 studies). The PDS was the only scale with validated psychometrics in the population of interest [95]. The majority of studies (*n* = 14; 58.3%) reported on past-month prevalence. A minority of studies used a 1- or 2-week time frame for assessment (*n* = 9; 36%), and only 2 studies (8%) assessed 1-year prevalence (see Table 1).

### Study quality

Overall, the methodology of the included studies had many strengths (see Table 2). Out of a maximum total of 8, quality scores ranged from 4 (50%) to 7 (87.5%) with an average score of 6.36 (79.5%) using Loney and colleagues (1998) [56]. Sampling methods and sample sizes were very strong. All but 2 of the 25 studies used random samples and described their sampling methods in detail. More than 70% of the studies used an unbiased sampling frame, such as census data, and sample sizes were large, ranging from 500 to 15,201 participants. Response rates were very high. One study did not report a response rate, and one had a response rate of only 64.3%, but for three-quarters of the studies response rates were >90%.

**Table 2:** Study quality indicators.

Country	Studies	1. Random sample or whole population	2. Unbiased sampling frame (i.e., census data)	3. Adequate sample size (>300 participants)	4. Measures were the standard^a^	5. Outcomes measured by unbiased assessors^b^	6. Adequate response rate (70%), refusers described	7. Confidence intervals, subgroup analysis	8. Study participants described	Total (max 8)	Percentage of 100%
**DRC**	**Johnson et al., 2010**	1	1	1	0.5	0.5	0.5	1	1	6.5	81.3%
**Ethiopia**	**Asnakew et al., 2019**	1	0	1	0.5	1	0.5	1	1	6	75.0%
**Kenya**	**Jenkins et al., 2015**	1	1	1	0.5	0.5	0.5	1	1	6.5	81.3%
**Liberia**	**Galea et al., 2010**	1	1	1	0.5	0.5	0.5	1	1	6.5	81.3%
	**Johnson et al., 2008**	1	1	1	0.5	0.5	0.5	1	1	6.5	81.3%
	**Vinck et al., 2013**	1	1	1	0.5	1	0.5	1	1	7	87.5%
**Nigeria**	**Gureje et al., 2006**	1	1	1	0.5	0.5	0.5	1	1	6.5	81.3%
**Rwanda**	**Fodor et al., 2015**	1	1	1	0.5	0.5	0.5	1	1	6.5	81.3%
	**Munyandamutsa et al., 2012**	1	0	1	0.5	0.5	0	1	1	5	62.5%
	**Pham et al., 2004**	1	0	1	0.5	0.5	0.5	1	1	5.5	68.8%
	**Rugema et al., 2015**	1	1	1	0.5	0.5	0.5	1	1	6.5	81.3%
**Sierra Leone**	**Betancourt et al., 2016**	1	1	1	0.5	1	0.5	1	1	7	87.5%
	**Jalloh et al., 2018**	1	1	1	0.5	1	0.5	1	1	7	87.5%
**South Africa**	**Herman et al., 2009**	1	1	1	0.5	0.5	0.5	1	1	6.5	81.3%
	**Machisa et al., 2017**	1	1	1	0.5	0.5	0.5	1	1	6.5	81.3%
	**Peltzer and Pengpid, 2019**	1	1	1	0.5	1	0.5	1	1	7	87.5%
	**Smit et al., 2006**	0.5	0	1	0.5	0	0	1	1	4	50.0%
	**Topper et al., 2015**	1	1	1	0.5	0.5	0.5	1	1	6.5	81.3%
**South Sudan**	**Ayazi et al., 2012**	1	1	1	0.5	1	0.5	1	1	7	87.5%
	**Ng et al., 2017**	0	0	1	0.5	1	0.5	1	1	5	62.5%
	**Roberts et al., 2009**	1	1	1	0.5	1	0.5	1	1	7	87.5%
**Uganda**	**Ertl et al., 2014**	1	0	1	1	1	0.5	1	1	6.5	81.3%
	**Mugisha et al., 2015**	1	0	1	0.5	1	1	1	1	6.5	81.3%
	**Pham et al., 2009**	1	0.5	1	0.5	1	0.5	1	1	6.5	81.3%
	**Vinck et al., 2007**	1	1	1	0.5	1	0.5	1	1	7	87.5%

^a^PTSD measurement tools consistent with generally accepted clinical/research criteria and tool validated during study or in population previously?

^b^Interviewers and their training described; internal reliability calculated.

**Abbreviations:** DRC, Democratic Republic of Congo; PTSD, posttraumatic stress disorder

Every study used accepted clinical or research tools for measuring PTSD. While most articles acknowledged that the tools had been validated elsewhere, of note, only one study in the meta-analysis used a tool that had been validated during the study in question or in the same study population previously, Ertl and colleagues (2014) in Uganda [79]. The assessors and their training to implement the instruments were well-described (all but 2 studies provided details about the interviewers); however, reliability was inconsistently reported. Over half (*n* = 14) of the studies ran and stated Cronbach's alpha for internal reliability, while the remaining 11 did not report it.

### Overall pooled prevalence of probable PTSD

**Fig 3:**
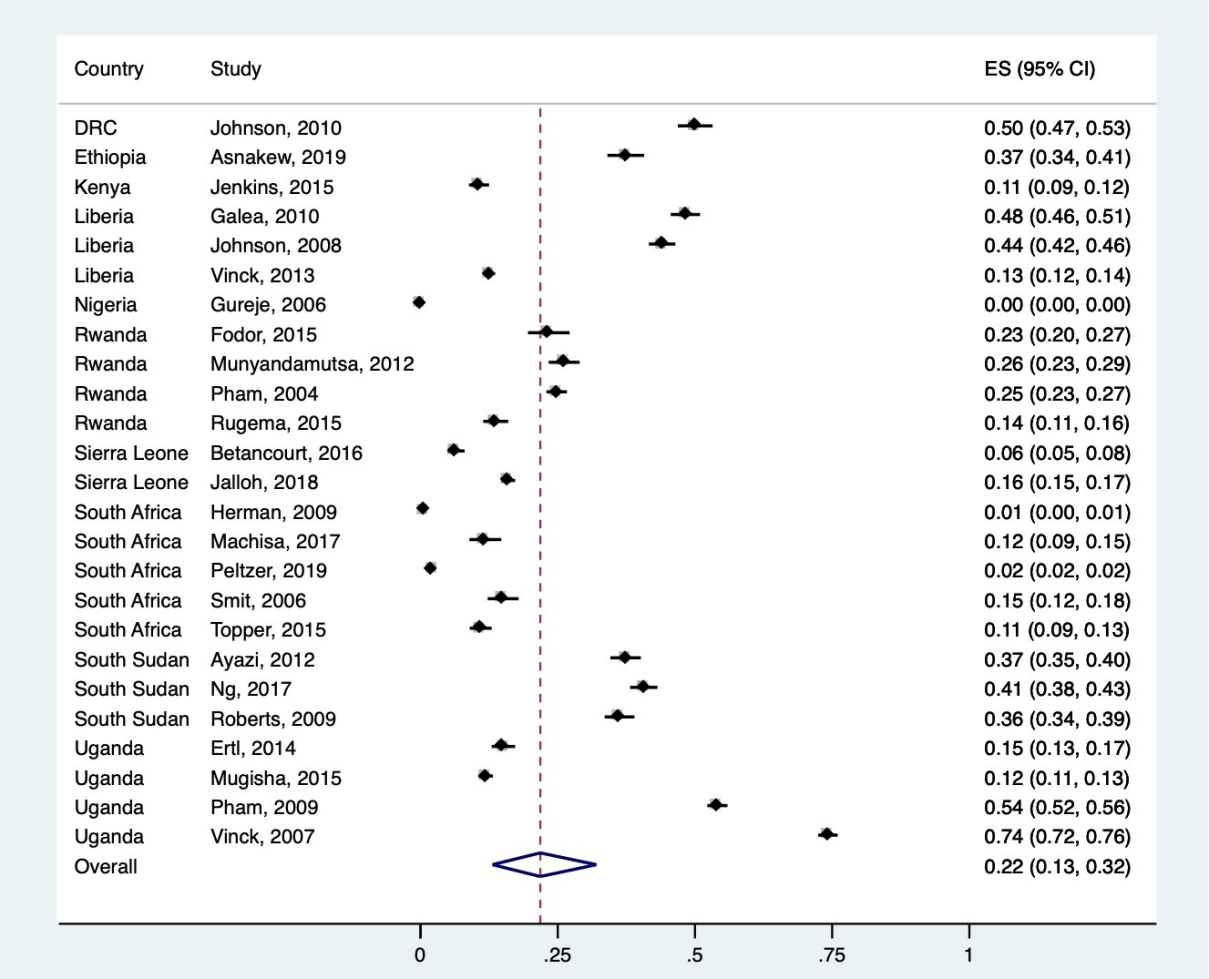
Overall prevalence estimates. CI, confidence interval; ES, effect size (proportion).

Prevalence estimates had reporting time frames of 1 week to 1 year, and estimates were highly variable, ranging from 0% (95% CI 0%–0%) to 74% (95% CI 72%–76%), and heterogeneous (Q = 18,326.70, df = 24, *p* < 0.001, *I*^2^ = 99.87%). Overall, pooled prevalence across all studies was 22% (95% CI 13%–32%) (see Fig 2 and Fig 3). Pooled estimates were recalculated only including the 20 studies that achieved at least an 80% quality score. The pooled estimate from the high-quality studies was 20% (95% CI 11%–32%). There was no association between study quality and prevalence (b = −0.001 [95% CI −0.11 to 0.11], *p* = 0.98).

### Pooled prevalence by sex

Five studies [71,79,84,96,97] did not report prevalence estimates by sex, and so they were not included in the analysis of prevalence by sex. Herman and colleagues (2009) [75] and Gureje and colleagues (2006) [98] did not report prevalence by sex but did report that there was no significant difference by sex, and so the overall mean score was included as the score for both men and women. Machisa and colleagues (2017) [85] only sampled women, and so the data from this study were included in the pooled analysis for women but not men.

Of the 19 studies that reported data comparing the rates of PTSD in men and women, 12 reported a significant difference (see Table 1). Of the 12 studies that reported a significant difference in the prevalence of PTSD in men compared to women, 10 reported higher rates of PTSD in women, and 2 reported higher rates of PTSD in men. There were no significant differences in the pooled prevalence of PTSD by sex (test of heterogeneity between groups = 0.42, df = 1, *p* = 0.52). The pooled prevalence estimate for females was 25% (95% CI 14%–39%). The pooled prevalence estimate for males was 20% (95% CI 10%–31%) (see S1 Fig). Post hoc meta-regression indicated that the lack of a significant difference in probable PTSD prevalence by sex persisted when predictors included conflict exposure, reporting time frame, and assessment tool type (b = −0.08 [95% CI −0.19 to 0.04], *p* = 0.18).

### Pooled prevalence by reporting time frame

The 2 WMH Survey studies [75,98] were the only ones to use a 1-year time frame. Because the study by Ayazi and colleagues [99] was the only study that utilized a 2-week time frame, it was grouped with those using a 1-week time frame when stratifying by assessment time frame, since this study used the HTQ [86], which has a 1-week–assessment time frame [86]. Across all studies, there were significant differences by assessment time frame (random heterogeneity test between subgroups = 79.61, df = 2, *p* < 0.001; see S2 Fig). However, this difference is explained by the 2 studies that assessed PTSD over a 1-year time frame. These 2 studies (Gureje and colleagues [98] and Herman and colleagues [75]) were part of the cross-national WMH Surveys [100] and were the only 2 studies to use the CIDI [94]. They each had pooled prevalence estimates of 0% (95% CI 0%–0%). In contrast, the pooled prevalence in the past week was 22% (95% CI 9%–38%), and the pooled prevalence in the past month was 27% (95% CI 16%–40%). There was no significant difference in the weekly versus monthly pooled prevalence (random heterogeneity test between subgroups = 0.28, df = 1, *p* ≤ 0.60). The overall pooled prevalence of probable current PTSD, defined as a period prevalence ranging from 1 week to 1 month, was 25% (95% CI 16%–36%).

### Pooled prevalence by screener versus diagnostic assessment tool

There was not a significant difference in the prevalence estimates between studies that used screening instruments (27% [95% CI 15%–41%]) compared to those using diagnostic structured and semi-structured interviews (14% [95% CI 4%–29%]; random test for heterogeneity between subgroups = 1.76, df = 1, *p* = 0.18).

### Pooled prevalence by exposure to war or armed conflict

**Fig 4:**
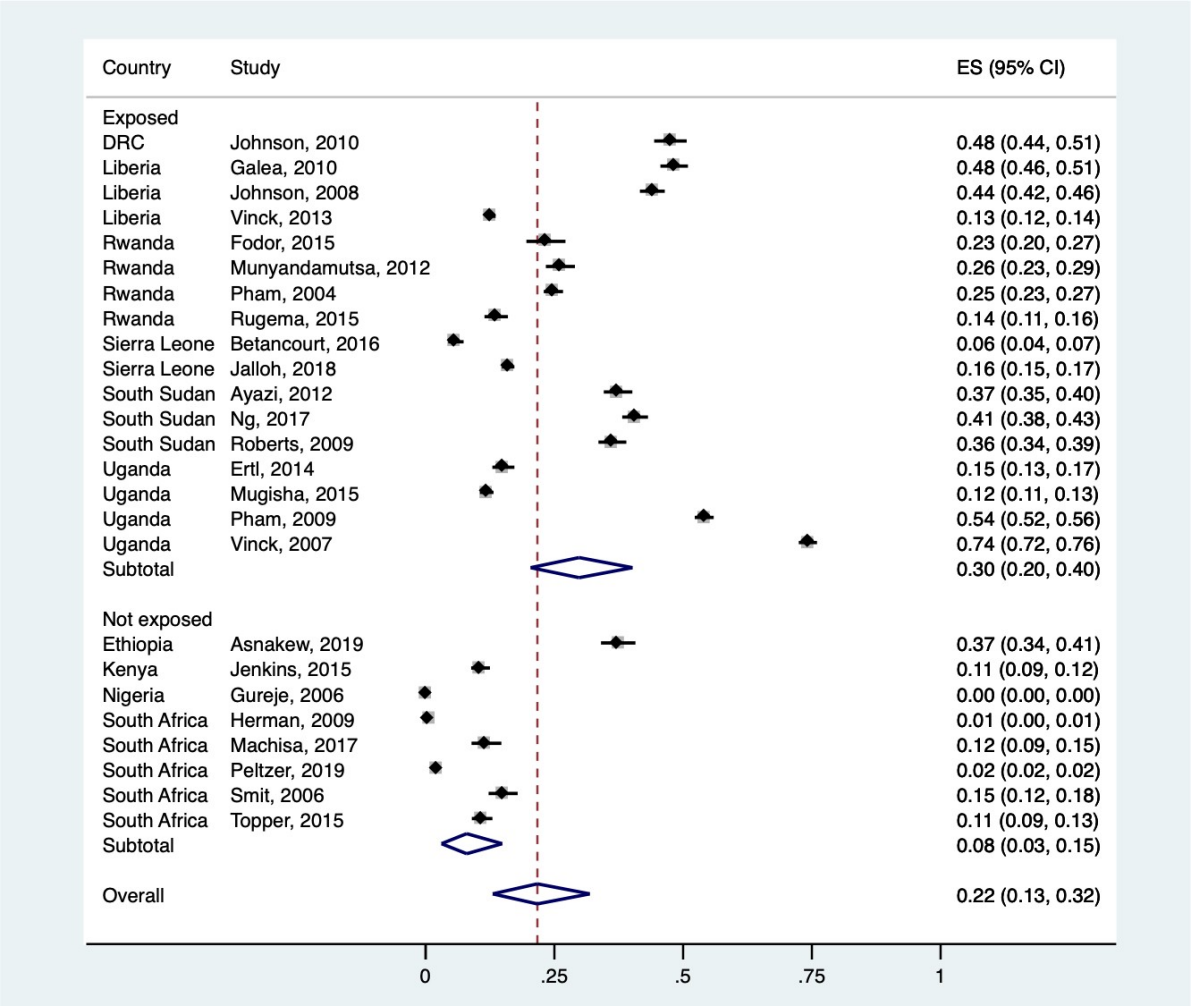
Prevalence estimates by exposure to mass-casualty war or armed conflict. Random test of heterogeneity between subgroups: 13.64, df = 1, *p* < 0.001. CI, confidence interval; ES, effect size (proportion).

There was a significant difference in pooled prevalence estimates between studies that were conducted in regions exposed to mass-casualty war or armed conflict at any point during the lifetime of the participants and those that were unexposed (random test for heterogeneity between subgroups = 13.64, df = 1, *p* = 0.01) (see Fig 4). The pooled prevalence estimate of studies from exposed regions was 30% (95% CI 20%–40%), while the estimate from unexposed regions was 8% (95% CI 3%–15%). Results of the adjusted *R*^2^ from a meta-regression indicated that conflict exposure accounted for 22.77% of the variance in the pooled prevalence estimates.

## Discussion

The goal of this review was to provide a more complete picture of the population-based data on the current prevalence of PTSD in SSA than is available from any single study. We identified 25 studies assessing 58,887 individuals in the SSA region. There were 4 key findings in the meta-analysis: (1) The overall pooled prevalence across all studies, including 2 studies that calculated a 1-year prevalence estimate, was 22% (95% CI 13%–32%). The pooled prevalence of current symptoms (defined as duration of 1 week to 1 month) consistent with a probable diagnosis of PTSD in SSA was 25% (95% CI 16%–36%). (2) Prevalence estimates of current symptoms across the individual studies were highly variable, ranging from 2% (95% CI 2%–2%) to 74% (95% CI 72%–76%). (3) There was no significant difference in the pooled prevalence of PTSD for men and women, for reporting time frame, or for whether the study used a screening versus diagnostic instrument. (4) Prevalence estimates across regions differed substantially by population-level exposure to war or armed conflict. We discuss these findings in more detail subsequently.

The high 22% prevalence of probable PTSD found in this meta-analysis seems to be driven in large part by the 30% prevalence of probable PTSD found across the 17 studies that were conducted on populations exposed to war and armed conflict. The finding that populations experiencing war or armed conflict within the lifetime of the study participants have higher probable PTSD prevalence is consistent with expectations. Countries with higher rates of conflict have populations exposed to higher levels of trauma and thus more PTSD symptoms. However, the prevalence of PTSD of 30% found in this meta-analysis still exceeds the rates found in 3 other meta-analyses in populations exposed to war of 15% [50], 26% [101], and 24% [51]. Moreover, the 8% prevalence of PTSD in non–war-exposed populations exceeds the 4% rate of PTSD prevalence found in other population-based cross-national studies [2], suggesting that there was still a substantial population-level burden of PTSD symptoms in non–conflict-affected countries, a burden that has often been overlooked. Taken together, these data suggest that PTSD symptoms and probable PTSD are common in SSA. The higher prevalence of PTSD found in this meta-analysis may be partially explained by studies finding that people living in SSA may be disproportionately affected by individual and population-level trauma exposure [8,10–12,14,102] and that the vast majority of people in SSA have very low access to mental health treatment [15,16]. These factors may result in a larger effect on the population burden of PTSD in SSA [22,23].

There was no consistent pattern in the results for sex differences in PTSD prevalence. While 10 of the 19 studies that reported on sex differences in PTSD prevalence reported that women had higher rates of probable PTSD, 7 studies reported no sex difference, and 2 studies reported that men had higher rates of probable PTSD. However, there was no difference in the overall pooled prevalence by sex. These findings suggest that sex may frequently be a critical variable to consider when understanding PTSD prevalence but that its explanatory power may vary by population context.

Due to large variability in prevalence estimates, there was not a significant difference in prevalence estimates between studies that used screening instruments compared to those using diagnostic structured and semi-structured interviews. However, the mean ratio of the difference between studies using screening instruments (27% [95% CI 15%–41%]) and those using diagnostic structured and semi-structured interviews (14% [95% CI 4%–29%]) was in line with the results of a meta-regression of prevalence of PTSD in countries experiencing war and armed conflict that found that symptom scales produced prevalence estimates that were 1.5 to 2 times higher than diagnostic tools [50]. The authors of the meta-regression suggest that this discrepancy may occur because symptom scales do not assess clinical significance or functional impairment and may therefore overestimate the prevalence of PTSD [50]. While the lack of data on functional impairment in symptom scales is certainly problematic, studies have found a strong correlation between elevated distress on symptom scales and functional impairment [103–105]. Indeed, subthreshold PTSD is associated with high distress and impairment and increased risk of suicidality globally [106]. Given that only 1 study used an instrument for PTSD that had been validated in the population of interest, we are unable to conclude whether symptom scales or diagnostic instruments may be under- or overestimating PTSD.

### Limitations

In producing pooled estimates, we encountered at least 4 limitations of the identified studies that limit our ability to make interpretations from our findings. First, only 1 study (Ertl et al., 2014 [78,79]) used a measure of PTSD symptoms whose reliability and validity had been assessed previously in the population of interest. PTSD symptoms are highly prevalent in the SSA countries where they have been studied. However, the lack of reliable and valid instruments used in the populations studied is a major limitation for both research and practice. In a 2018 review of qualitative studies examining post-trauma symptoms across cultural contexts, most study participants reported symptoms consistent with Western diagnostic criteria but also reported a number of symptoms not captured in those diagnostic classifications [107], supporting the need for local contextualization of measures. Measures that are not locally contextualized and valid may result in substantial under- or overreporting of trauma exposure and PTSD symptoms. For example, a study of Zulu-speaking participants in northeastern KwaZulu-Natal, South Africa found that the PTSD section of the Structured Clinical Interview for Diagnostic and Statistical Manual (DSM) Disorders, Axis I, Research Version (SCID-I RV) [108] undercounted participants who had been exposed to traumatic events by almost 20% compared to a Zulu Culture-Specific Trauma Experience Questionnaire [109]. Pragmatic and feasible approaches to cultural and contextual validation of measures of post-trauma symptoms in challenging settings have been described [110] and need to become more widely employed prior to undertaking population surveys. Rigorous epidemiological research to examine the predictive validity of PTSD constructs in diverse settings is also an important priority.

Second, although data were available from a relatively large number of participants, the studies only represent data from 10 of the 48 SSA countries, and only 6 studies provided national-level data. There is a complete absence of national or regional population-based data on PTSD from more than 80% of SSA countries, including those affected by ongoing fragile and conflict-affected situations, such as Central African Republic, Mali, and Somalia. Given the enormous heterogeneity expected not only across the continent but also within countries and regions, this review cannot speak to rates of PTSD in any of these other regions. Thus, substantial gaps in our knowledge of PTSD prevalence in the majority of SSA remain.

These disparities in PTSD prevalence data parallel the lack of mental health data and services broadly in SSA [1]. Despite the enormous burden that mental disorders are projected to pose in SSA by 2050, they remain a low priority in terms of policy initiatives and research funding [111]. Even with political will and support, many countries may have difficulty meeting identified mental health needs given limited resources and competing priorities. Indeed, although 72% of countries in the Africa region reported that they had a stand-alone mental health policy, only 27% had allocated resources towards implementing that plan [112].

Third, as noted previously, there was high heterogeneity in the prevalence estimates with an *I*^2^ of more than 99%. We were therefore unable to statistically assess the risk of publication bias in this meta-analysis because none of the publication bias methods provide accurate results with more than moderate levels of heterogeneity (i.e., *I*^2^ < 50%) [113]. We are therefore unable to provide insight into the level of publication bias that may be present in these results.

Finally, the goal of this review was to focus on adult PTSD, and we excluded studies that focused exclusively or primarily on participants who are younger than 15 years old, which is the youngest age that the World Bank considers part of the “working age population” and is the youngest age of majority in countries throughout the world. All of the identified studies had participants whose mean age was 18 years old or older. However, 5 studies [70,79,82,83] included individuals who were younger than 18 years old, and therefore some of the data come from adolescents. However, when these studies were removed from the analyses, the results were unchanged.

In addition, there are several limitations in the methodology of our review that should be noted. Our inclusion criteria required that studies have at least 450 participants, use a probabilistic sampling procedure, and report a quantitative estimate of PTSD. As a result, we excluded smaller studies, those that focused on key populations, and those that reported only continuous measures of PTSD symptoms. Our review does not, therefore, speak to subthreshold PTSD, PTSD in specific populations (e.g. refugees, people living with HIV, students, or combatants), or nuances in context that may impact PTSD presentation and prevalence, such as IDPs currently living in conflict zones compared to those living in safe environments.

### Disparities between our pooled estimates and WMH surveys

The pooled prevalence of probable PTSD in this systematic review (computed from 1 week to 1 year prevalence rates) is extremely high compared to the prevalence reported by a 2017 summary of data from the WMH surveys. For example Karam and colleagues (2014) [4] reported a WMH 12-month survey prevalence of 1.1% ranging from 3.8% in Northern Ireland to a low of 0.2–0.3% in Beijing and Shanghai in the People’s Republic of China, 0.3% in Colombia, and 0.3% in Mexico [4]. Indeed, the current PTSD pooled prevalence found in this study far exceeds the 3.9% lifetime rate found in the WMH surveys [2]. There are at least 4 possible reasons for the disparity found between the pooled PTSD prevalence estimates identified in this meta-analysis and the prevalence estimates found in the WMH surveys. First, although our review reports data from only 10 countries, 6 of those countries have experienced war and armed conflict during the lifetime of the participants. Very few such countries are included in the WMH surveys, with Iraq and Lebanon being notable exceptions [4]. However, even in Iraq and Lebanon, the prevalence of PTSD diagnosis is very low compared with the estimates of prevalence observed in SSA. Second, many of the studies in our pooled estimate specifically focused on PTSD assessment as a primary aim of the study. The WMH survey, in contrast, aims to document the population burden of all mental and behavioral disorders globally without one particular focus. In addition, in most cases the survey is administered in 2 stages, and PTSD is only included in the second stage of the survey [114]. Third, there are specific assessment nuances of the CIDI [100] (WMH survey studies were the only ones that used the CIDI) that may have impacted PTSD symptom report. First, in this review, the CIDI was the only diagnostic interview that was lay administered. Another key issue with the CIDI, compared to other instruments used in most of the studies, is the skip out related to reporting trauma. That is, PTSD is only assessed in persons who reported a qualifying trauma. Thus, if a participant does not report a trauma, either because their trauma is not on the list queried or because the person chooses not to report such sensitive information, then these participants do not get assessed for PTSD [114]. As a result, PTSD symptoms may go undetected. This is particularly problematic when the trauma events have not been culturally and contextually validated [109]. There is some data to suggest that the results of trauma may be underreported in the WMH surveys. For example, the lifetime exposure of sexual violence in the South African WMH Survey found a prevalence rate of rape of 2%; however, other epidemiological surveys from South Africa have found lifetime rates of rape between 4.5% and 12% [115].

Fourth, there may be differences in the way that individuals from different countries, communities, and cultures respond to items, which may result in under- or overreporting of symptoms. The CIDI, like many highly structured interviews, applies an algorithm of combinations of types of symptoms following the Diagnostic and Statistical Manual version 4 (DSM-IV) [116] in order to produce a diagnosis. The algorithms, like the diagnostic criteria in DSM-IV, are derived predominantly from Western populations, which give weight to the core symptoms that are known to have salience in those settings but not necessarily in other settings. Concerns about the cross-cultural applicability of the CIDI have been reported in Nepal [117], Ethiopia [118], and American Indian Reservation populations [119]. Indeed, a study that used latent-class analysis to reexamine the depression results of the WMH surveys from the US, New Zealand, South Africa, and Nigeria found that participants in Nigeria and South Africa who endorsed the screening questions endorsed more severe depression symptoms than participants from the US and New Zealand who endorsed the screening questions [120]. When these differential patterns of responding were taken into account, the prevalence estimate for depression in Nigeria was highest (22%), whereas the results of the Nigeria WMH Survey found that it had the lowest rate of depression (3%). Studies are needed that examine the reliability and validity of the diagnostic criteria of PTSD as well as widely used assessments such as the CIDI in SSA populations.

## Conclusions and directions for future study

Methodological limitations of the extant literature as described earlier lead us to conclude that our pooled estimate should be interpreted with caution. Given limited information on the reliability and validity of the assessment tools used and the lack of data available from most countries in the region, more work is needed before strong conclusions can be made about the population burden of PTSD in SSA. Furthermore, the identified studies provide little or no evidence regarding the proportion of the population requiring specific levels of intervention and therefore do little to inform service planning.

However, even given these limitations, our findings suggest that health systems in SSA need to improve the identification of and access to treatment for persons with PTSD. The percentage of individuals with PTSD seeking treatment is globally low and is most strikingly low in countries of low to lower-middle income and of upper-middle income [2]. Improving detection of trauma and PTSD in primary care may be an efficient strategy, given that globally, people with PTSD are more likely to seek care in general health settings than in specialty mental health clinics [2]. However, major efforts to scale up mental healthcare, such as the Programme for Improving Mental Health Care (PRIME) [121]—which is focused on improving mental healthcare in nonhumanitarian settings in several low- and middle- income countries through integration with maternal and primary care—have not, to date, included PTSD as a target disorder. This is likely, in part, due to a lack of data on the prevalence of PTSD and the burden it poses in these settings. Moreover, misconceptions about PTSD remain. For example, PTSD is more widely recognized as a problem for refugees, in high-conflict situations, and in humanitarian crises. PTSD is included in the Mental Health Gap Action Programme (mhGAP) Intervention Guide for humanitarian settings but not yet included in the primary mhGAP [122,123]. However, epidemiologic data suggest that, globally, sexual violence in the context of intimate partnerships is responsible for the largest burden of PTSD [5]. Thus, much foundational epidemiological work remains to be done to document the burden of PTSD in SSA. This work will be critical to inform policy, research, and treatment; above all, it will be critical to address issues of access to care and decrease the burden of PTSD in SSA.

## Supporting information

S1 PRISMA Checklist (DOC)

S1 Fig Pooled prevalence by sex. Random test of herterogeneity between subgroups: 0.42, df = 1, *p* = 0.052. CI, confidence interval; ES, effect size (proportion)
(TIFF)

S2 Fig Pooled prevalence by reporting time frame. Random test of heterogeneity between subgroups: 79.61, df = 2, *p* < 0.001. CI, confidence interval; ES = efect size (proportion)
(TIFF)

## Abbreviations

CIDI: Composite International Diagnostic Interview
DRC: Democratic Republic of Congo
DSM-IV: Diagnostic and Statistical Manual version 4
DTS: Davidson Trauma Scale
EVD: Ebola virus disease
HTQ: Harvard Trauma Questionnaire
HTQ-R: Harvard Trauma Questionnaire-Revised
ICTR: United Nations’ International Criminal Tribunal for Rwanda
IDP: internally displaced person
IES-6: Impact of Event Scale-6
MeSH: medical subject heading
mhGAP: Mental Health Gap Action Programme
MINI: Mini International Neuropsychiatric Interview
PCL-C: PTSD Checklist-Civilian Version
PDS: Posttraumatic Stress Diagnostic Scale
PRIME: Programme for Improving Mental Health Care
PRISMA: Preferred Reporting Items for Systematic Reviews and Meta-Analyses
PROSPERO: International Prospective Register of Systematic Reviews
PSS-I: PTSD Symptom Scale Interview
PTSD: posttraumatic stress disorder
SCID: Structured Clinical Interview for DSM Disorders, Axis I, Research Version
SSA: sub-Saharan Africa
TSQ: Trauma Screening Questionnaire
WMH: World Mental Health
