# Correction: National and regional prevalence of posttraumatic stress disorder in sub-Saharan Africa: A systematic review and meta-analysis

**DOI:** 10.1371/journal.pmed.1003312

**Published:** 2020-07-24

**Authors:** Lauren C. Ng, Anne Stevenson, Sreeja S. Kalapurakkel, Charlotte Hanlon, Soraya Seedat, Boniface Harerimana, Bonginkosi Chiliza, Karestan C. Koenen

In the byline, some affiliation numbers are attributed incorrectly to some authors. The byline and affiliation list should read:

Lauren C. Ng^1^*, Anne Stevenson^2^, Sreeja S. Kalapurakkel^3^, Charlotte Hanlon^4^, Soraya Seedat^5^, Boniface Harerimana^6,7^, Bonginkosi Chiliza^8^, and Karestan C. Koenen^9^

**1** Department of Psychology, University of California Los Angeles, Los Angeles, California, United States of America, **2** Department of Epidemiology, Harvard T.H. Chan School of Public Health, Boston, Massachusetts, United States of America, **3** Duke University Global Health Institute, Durham, North Carolina, United States of America, **4** Centre for Global Mental Health, Health Service and Population Research, Department Institute of Psychiatry, Psychology and Neuroscience King’s College, London, United Kingdom, **5** Department of Psychiatry, Stellenbosch University, Cape Town, South Africa, **6** Faculty of Health Sciences, Western University, London, Ontario, Canada, **7** College of Medicine and Health Sciences, University of Rwanda, Kigali, Rwanda, **8** Department of Psychiatry, Nelson R. Mandela School of Clinical Medicine, University of KwaZulu-Natal, Durban, South Africa, **9** Department of Epidemiology, Harvard T.H. Chan School of Public Health, Boston, Massachusetts, United States of America
