# Supplementary material for: National and regional prevalence of posttraumatic stress disorder in sub-Saharan Africa: A systematic review and meta-analysis
Source: PLoS Med. 2020 May 15;17(5):e1003090. doi: 10.1371/journal.pmed.1003090 (PMC7228043; doi:10.1371/journal.pmed.1003090)
Supplement: S1 PRISMA Checklist — (DOC) [file pmed.1003090.s001.doc]

| **Section/topic** | **#** | **Checklist item** | **Reported on Section + Paragraph #** |
| --- | --- | --- | --- |
| **TITLE** | | |  |
| Title | 1 | Identify the report as a systematic review, meta-analysis, or both.  National and Regional Prevalence of Posttraumatic Stress Disorder in Sub-Saharan Africa: A Systematic Review and Meta-Analysis | Title, 1 |
| **ABSTRACT** | | |  |
| Structured summary | 2 | Provide a structured summary including, as applicable: background; objectives; data sources; study eligibility criteria, participants, and interventions; study appraisal and synthesis methods; results; limitations; conclusions and implications of key findings; systematic review registration number.  Background:  People living in sub-Saharan Africa (SSA) are disproportionately exposed to trauma exposure and may be at increased risk for posttraumatic stress disorder (PTSD). However, a dearth of population-level representative data from SSA is a barrier to assessing PTSD. This manuscript sought to calculate pooled PTSD prevalence estimates from nationally and regionally representative surveys in SSA.  Methods and findings:  The search was conducted in PubMed, Embase, PsycINFO, and PTSDpubs and was last run between October 18, 2019 and November 11, 2019. We included studies that were published in peer-reviewed journals; used probabilistic sampling methods and systematic PTSD assessments; and included > 450 participants who were current residents of a SSA country, at least 50% of whom were between 15-65 years. The primary outcomes were point prevalence estimates of PTSD across all studies, and then within subgroups. The protocol was registered with PROSPERO (# CRD42016029441). Out of 2825 unique articles reviewed, 25 studies including a total of 58,887 eligible participants (54% female) in ten out of the 48 countries in SSA were identified. Most studies enrolled any adult aged 18 years or older. However, some studies only enrolled specific age brackets or persons as young as 15 years old. Six studies were national surveys and 19 were regional.  There were four key findings in the meta-analysis: (1) The overall pooled prevalence of probable PTSD was 22% (95% CI = 13% - 32%), while the current prevalence, defined as one week to one month, was 25% (95% CI: 16% to 36%); (2) Prevalence estimates were highly variable, ranging from 0% (95% CI: 0%-0%) to 74% (95% CI: 72%-76%); (3) Conflict unexposed regions had a pooled prevalence of probable PTSD of 8% (95% CI: 3%-15%) while conflict exposed regions had a pooled prevalence of probable PTSD of 30% (95% CI: 21%-40%; *p*<.001); (4) There was no significant difference in the pooled prevalence of PTSD for men and women. The primary limitations of our methodology are our exclusion of studies published in languages other than English, French and Portuguese, smaller studies, those that focused on key populations, those that reported only on continuous measures of PTSD symptoms, and unpublished or non-peer reviewed studies.  Conclusions:  In this study, PTSD symptoms consistent with a probable diagnosis were found to be common in sub-Saharan Africa, especially in regions exposed to armed conflict. However, these studies only represent data from 10 of the 48 SSA countries, and only six studies provided national level data. Given the enormous heterogeneity expected across the continent, and also within countries and regions, this review cannot speak to rates of PTSD in any regions not included in this review. Thus, substantial gaps in our knowledge of PTSD prevalence in SSA remain. More research on population level prevalence is needed to determine the burden of trauma symptoms and PTSD in SSA and to identify acceptable and feasible approaches to address this burden given limited mental health care resources. | Abstract |
| **INTRODUCTION** | | |  |
| Rationale | 3 | Describe the rationale for the review in the context of what is already known.  Mental and substance use disorders account for 23% of years lost to disability, making them the leading cause of disability worldwide (1). Posttraumatic stress disorder (PTSD) is a large contributor to the global burden of disease and is estimated to affect almost 4% of the world’s population (2). PTSD persists for over a year in 50% of all cases (2) and often leads to substantial declines in functioning and productivity (3). National and regional data on prevalence are used to develop policies and action plans for addressing PTSD and other related disorders. Recently much of the data on global and national estimates of PTSD has come from the World Health Organization’s World Mental Health (WMH) surveys of the cross-national prevalence of PTSD in 26 countries (2, 4, 5). The WMH surveys collected representative population data across the world using structured diagnostic measures to assess PTSD, which allowed for the calculation of global population prevalence estimates of PTSD (2). However, the WMH surveys only included one national survey and one regional survey from sub-Saharan Africa (SSA); in addition, the one national estimate was from South Africa, one of the few upper-middle income countries in SSA (2). The limited number of countries from SSA contributing data on PTSD in the WMH surveys is consistent with the lack of population mental health data from SSA generally (6, 7).  People living in SSA may be disproportionately affected by individual and population level trauma exposure. Indeed, research from the World Health Organization has found that the lifetime prevalence of road traffic deaths (8) and of reported intimate partner violence and/or non-partner sexual violence are highest in the Africa region (9, 10). In addition, although other regions of the world experience more natural disasters, the great majority of countries most vulnerable to natural disasters are in SSA (11). SSA has also been disproportionately affected by war and armed conflicts, many of which have been ongoing for years, if not decades (12). In 2019, 20 countries in SSA were classified by the World Bank as hosting fragile and conflict-affected situations, which represents more than 50% of the fragile and conflict-affected countries globally (13). Moreover, the legacy of violence, loss, and historical trauma inflicted on the people in SSA through colonization may contribute to high rates of posttraumatic stress (14).  Increased exposure to traumatic life experiences in SSA is compounded by very low rates of access to mental health treatment (15, 16). Of the 48 countries in SSA (17), 24 (50%) are low-income countries, 18 (37.5%) are lower-middle-income countries, and the remaining six (12.5%) are upper middle-income countries (18). It is estimated that the gap between those who need mental health care and those who receive it often exceeds 90% in low-income countries for most mental disorders (19-21). It is estimated that 77% of people with PTSD in lower-middle-income countries have not received treatment (2). Given that the majority of countries in Africa are low-income or lower-middle-income, it is likely that the vast majority of people with PTSD in SSA will never receive treatment and are at high risk for chronic symptoms. Repeated and prolonged exposure to violence, armed conflict, and mass-casualty events, combined with a lack of access to mental health treatment (15, 16), may result in an outsized effect on the population burden of PTSD in SSA (22, 23). | Introduction, 1-3 |
| Objectives | 4 | Provide an explicit statement of questions being addressed with reference to participants, interventions, comparisons, outcomes, and study design (PICOS).  The goal of this meta-analysis was to synthesize the existing data on the population prevalence of PTSD in SSA. While many studies of PTSD have been conducted in SSA, most of these studies derived their estimates from non-representative samples or specific populations such as refugees or internally displaced persons (24-30), patients (31-35), parents (36-40), or students (41-49). While studies have summarized PTSD prevalence in conflict-affected populations including those in SSA (50, 51), PTSD occurs in response to a wide range of traumas (both interpersonal and non-interpersonal) that occur in non-conflict settings (e.g. car accidents, sexual assault). Population representative epidemiologic data is critical to understand the burden of PTSD in SSA and develop national and regional policies to address that burden. To our knowledge this is the first meta-analysis to summarize the data on population-based point prevalence of PTSD in SSA across all settings. The objectives of this paper were to a) conduct a systematic review and meta-analysis of the prevalence of PTSD from representative national or regional studies, and b) explore the association between gender, population-level exposure to armed conflict, and pooled prevalence of PTSD. | Introduction, Aims, 1 |
| **METHODS** | | |  |
| Protocol and registration | 5 | Indicate if a review protocol exists, if and where it can be accessed (e.g., Web address), and, if available, provide registration information including registration number.  This systematic review was registered in the PROSPERO International Prospective Register of Systematic Reviews on December 1, 2016 and updated on June 21, 2019 (registration number CRD42016029441; <https://www.crd.york.ac.uk/prospero/display_record.php?RecordID=29441&VersionID=52136>). | Methods, 7 |
| Eligibility criteria | 6 | Specify study characteristics (e.g., PICOS, length of follow-up) and report characteristics (e.g., years considered, language, publication status) used as criteria for eligibility, giving rationale.  Study inclusion criteria:   1. Participants were current residents of a country in SSA (e.g., citizens, permanent residents, internally displaced persons (IDPs), or refugees or immigrants who had resided in the country for at least six months). 2. In order to capture studies that focused primarily on adults, at least 50% of participants had to be between 15 and 65 years of age, which is defined as the “working age population” by the World Bank (52). 3. PTSD data were reported for at least 450 participants. We selected *a priori* a sample size cut off to ensure that we would have enough power to detect prevalence estimates lower than 3.9%, which is the mean lifetime PTSD prevalence across the WMH surveys (2). A minimum sample size requirement of 450 would allow us to identify prevalence as low as 3.3% with a precision of 1.65% within each study or region (53). 4. Studies that used a systematic method of classifying participants as those with or without PTSD. Studies could be included if they used structured or semi-structured interviews, either administered by trained lay interviewers or clinicians, to provide a diagnosis or used a symptom checklist or diagnostic assessment and applied a cut point to classify people as having PTSD or not having PTSD. In addition, we included studies that assessed cultural idioms of distress that were clearly related to symptoms developed in response to a stressful or traumatic event (54). These are referred to as “post trauma reaction syndromes.” For the purposes of this paper, any participants who were either diagnosed with PTSD following an interview or who scored above a cut-point criterion set by the study authors were considered to have symptoms consistent with a diagnosis of PTSD. 5. Studies that employed probabilistic procedures to obtain nationally or regionally representative samples. Representative samples that were limited to a specific gender were also included; 6. Articles that were written in English, French or Portuguese, which covers most of the scientific literature from SSA; 7. Studies that were published in a peer-reviewed journal with no journal date restrictions. 8. If studies included a mixture of eligible and non-eligible participants and data from the group meeting inclusion criteria could be disaggregated, the data that met inclusion criteria were included in the review. | Methods, Search Strategy and Selection Criteria, 1 |
| Information sources | 7 | Describe all information sources (e.g., databases with dates of coverage, contact with study authors to identify additional studies) in the search and date last searched.  The search strategy was developed and initially conducted in PubMed between February 22, 2016 and August 1, 2017. The search was expanded to four databases and rerun using PubMed, Embase, PsycINFO, and PTSDpubs between October 18, 2019 and November 11, 2019. Search terms were “[Name of SSA Country] AND PTSD (in any field).” The search was run for each country in SSA. Using Kenya as an example, PubMed automatically generates the following search when these terms are entered: ("Kenya"[MeSH Terms] OR "Kenya"[All Fields]) AND ("stress disorders, post-traumatic"[MeSH Terms] OR ("stress"[All Fields] AND "disorders"[All Fields] AND "post-traumatic"[All Fields]) OR "post-traumatic stress disorders"[All Fields] OR "ptsd"[All Fields]). The full texts of articles that appeared to meet inclusion criteria based on the abstract were downloaded and references were reviewed to identify additional papers that might meet the eligibility criteria. | Methods, Search Strategy and Selection Criteria, 2 |
| Search | 8 | Present full electronic search strategy for at least one database, including any limits used, such that it could be repeated. | See item 7 above |
| Study selection | 9 | State the process for selecting studies (i.e., screening, eligibility, included in systematic review, and, if applicable, included in the meta-analysis).  Articles were identified through the PubMed, Embase, PsycINFO, and PTSDpubs search and by reviewing references from the identified papers. Seven fields were then extracted from each paper and entered into a Google Docs spreadsheet: article title, year, first author, journal, search date, researcher conducting the search, and abstract.  Duplicate entries were flagged and filtered out of the spreadsheet.  To identify eligible studies from the remaining articles, the full-text of each article was reviewed and one coder entered information related to each inclusion and exclusion criteria into the database. A second coder independently reviewed each of the inclusion and exclusion criteria to assess concordance. Disagreements about study eligibility were reviewed and discussed by the research team and final decisions were made by consensus.  The abstracts of non-English articles were reviewed by native speakers of the article language to determine eligibility, and if the abstract was deemed eligible, the full article was reviewed by the native speaker and also underwent targeted translation into English to enable accurate data extractions by a second coder. Studies that were not related to the eligibility criteria (e.g., a commentary or a study describing physical trauma instead of psychological trauma) were flagged and removed. For studies where it was unclear if they met eligibility criteria, the first author (L.N.) emailed the authors of the study to clarify. | Methods, 4 |
| Data collection process | 10 | Describe method of data extraction from reports (e.g., piloted forms, independently, in duplicate) and any processes for obtaining and confirming data from investigators.  Study quality was assessed using a standardized tool for observational studies (56). Loney et al. 1998 was selected against a range of checklists and scales based on its applicability to prevalence studies, validation, reliability, and clear methodology for rating studies (57). The tool contains eight items, each with a possible score of 1, with a maximum total score of 8.  In implementing the quality assessment tool, we operationalized four of the questions on the Loney tool to best suit the purposes of this systematic review and meta-analysis: 1. On the first question (“Are the study design and sampling method appropriate for the research question?”), a full point was given if the study was a whole population or a random sample and the authors described the process in enough detail to support the statement from region down to the randomization of the individual selected to participate. Studies received a zero if they were not the whole population or a random sample, or a half point if the study design was described as a “random sample”, but there was not enough evidence to confirm this. 2. On the fourth question (“Are objective, suitable and standard criteria used for measurement of the health outcome?”), we defined this as “PTSD measurement tools which are consistent with accepted clinical/research criteria and validated in the population of interest.” If the battery was an acceptable clinical/research tool, but had not been validated during the study or previously in the population of interest, it received a half point. 3. On the fifth question (“Is the health outcome measured in an unbiased fashion?”), we adapted this to make it more appropriate our study framework. The original tool recommends blinding interviewers to the purpose of the study in some cases. We did not feel this was applicable in the context of prevalence studies of PTSD in SSA as most teams employed interviewers to conduct the batteries and trained them specifically on research methods in order to implement the study. Papers received a full point on this question if they described the assessors, the training that they received to conduct the interviews, and internal reliability was calculated. If only one of these items was stated in the paper, the question was scored as a half point. 4. On question six (“Is the response rate adequate? Are the refusers described?”), papers received a full point if the response rate was at least 70% and if the authors described any demographic details about the refusers. Studies scored a zero if they did not meet the response rate threshold and did not describe the refusers, or a half point if they did one or the other. All other items on the Loney tool were scored as either 0 or 1 with no half points. The association between study quality and prevalence estimate was assessed using a meta-regression analysis.  Data were extracted from eligible articles by L.N. and all extractions were reviewed and confirmed by at least one other researcher. The primary outcomes of interest were the point prevalence estimates of PTSD. In addition, information on the number, age, and gender of participants, population level trauma exposure, sampling procedures, and the language, translation, validity, and reliability of the PTSD assessment tools were also extracted. | Methods, 5,6, and Data Analysis, 1 |
| Data items | 11 | List and define all variables for which data were sought (e.g., PICOS, funding sources) and any assumptions and simplifications made.  The primary outcomes of interest were the point prevalence estimates of PTSD. In addition, information on the number, age, and gender of participants, population level trauma exposure, sampling procedures, and the language, translation, validity, and reliability of the PTSD assessment tools were also extracted. | Methods, Data Analysis, 1-4 |
| Risk of bias in individual studies | 12 | Describe methods used for assessing risk of bias of individual studies (including specification of whether this was done at the study or outcome level), and how this information is to be used in any data synthesis.  Third, as noted above, there was high heterogeneity in the prevalence estimates with an I^2^ of more than 99%. We were therefore unable to statistically assess the risk of publication bias in this meta-analysis because none of the publication bias methods provide accurate results with more than moderate levels of heterogeneity (i.e. I^2^<50%) (123). We are therefore unable to provide insight into the level of publication bias that may be present in these results. | See item 10 about study quality  and Discussion, Limitations, 4 |
| Summary measures | 13 | State the principal summary measures (e.g., risk ratio, difference in means).  Pooled prevalence estimates were calculated across all studies, and then within subgroups including by gender, assessment time frame (i.e., one week, one month, one year), use of a screening or diagnostic measure, and whether populations were affected or not-affected by mass-casualty war or armed conflict. | Methods, Data Analysis, 2 |
| Synthesis of results | 14 | Describe the methods of handling data and combining results of studies, if done, including measures of consistency (e.g., I^2^) for each meta-analysis.  Pooled estimates were calculated using the Stata version 14.2 (59) metaprop (60) command, which allows for the inclusion of all studies, including those with 0% or 100% prevalence proportions. Metaprop was run using a) a random-effects model which assumes that differences in prevalence estimates are not solely due to sampling error, b) the exact confidence intervals, and c) the Freeman-Tukey double arcsine transformation to normalize the prevalence estimates prior to pooling (60). Q and I^2 were calculated to assess heterogeneity across all studies and within and between subgroups (61). Finally, a meta-regression with all a priori defined subgroups in which significant differences in pooled prevalence were identified was run using the Stata metareg command (62). | Methods, Data Analysis, 2 |

Page 1 of 2

| **Section/topic** | **#** | **Checklist item** | **Reported on page #** |
| --- | --- | --- | --- |
| Risk of bias across studies | 15 | Specify any assessment of risk of bias that may affect the cumulative evidence (e.g., publication bias, selective reporting within studies). | See item 12 above. |
| Additional analyses | 16 | Describe methods of additional analyses (e.g., sensitivity or subgroup analyses, meta-regression), if done, indicating which were pre-specified.  The association between study quality and prevalence estimate was assessed using a meta-regression analysis.  Pooled prevalence estimates were calculated across all studies, and then within subgroups including by gender, assessment time frame (i.e., one week, one month, one year), use of a screening or diagnostic measure, and whether populations were affected or not-affected by mass-casualty war or armed conflict. War or armed conflict was defined using the international humanitarian law definition, in which armed conflict occurs between organized armed groups, governmental or non-governmental(58). Pooled prevalences at the national and regional level were calculated to produce a heat map of available PTSD estimates in SSA (see Figure 2). Pooled estimates were calculated using the Stata version 14.2 (59) metaprop (60) command, which allows for the inclusion of all studies, including those with 0% or 100% prevalence proportions. Metaprop was run using a) a random-effects model which assumes that differences in prevalence estimates are not solely due to sampling error, b) the exact confidence intervals, and c) the Freeman-Tukey double arcsine transformation to normalize the prevalence estimates prior to pooling (60). Q and I^2 were calculated to assess heterogeneity across all studies and within and between subgroups (61). Finally, a meta-regression with all a priori defined subgroups in which significant differences in pooled prevalence were identified was run using the Stata metareg command (62). | Methods, 6  Methods, Data Analysis, 2 |
| **RESULTS** | | |  |
| Study selection | 17 | Give numbers of studies screened, assessed for eligibility, and included in the review, with reasons for exclusions at each stage, ideally with a flow diagram.  See **Figure 1: Study selection flow diagram** | **Figure 1: Study selection flow diagram** |
| Study characteristics | 18 | For each study, present characteristics for which data were extracted (e.g., study size, PICOS, follow-up period) and provide the citations.  See **Table 1. Identified studies** | **Table 1. Identified studies** |
| Risk of bias within studies | 19 | Present data on risk of bias of each study and, if available, any outcome level assessment (see item 12).  **Study quality**  Overall, the methodology of the included studies had many strengths (see Table 2). Out of a maximum total of 8, quality scores ranged from 4 (50%) to 7 (87.5%) with an average score of 6.36 (79.5%) using Loney et al., 1998 (56). Sampling methods and sample sizes were very strong. All but two of the 25 studies used random samples and described their sampling methods in detail. More than 70% of the studies used an unbiased sampling frame, such as census data, and sample sizes were large, ranging from 500 to 15,201 participants. Response rates were very high. One study did not report a response rate and one had a response rate of only 64.3%, but for three-quarters of the studies response rates were > 90%. | Results, Study Quality, 1  And  **Table 2. Study quality indicators** |
| Results of individual studies | 20 | For all outcomes considered (benefits or harms), present, for each study: (a) simple summary data for each intervention group (b) effect estimates and confidence intervals, ideally with a forest plot.  See Figure 3. Overall prevalence estimates  Figure 4. Prevalence estimates by exposure to mass casualty war or armed conflict  Supplementary Figure 1. Pooled prevalence by gender  Supplementary Figure 2: Pooled prevalence by reporting time frame | Figures 3, 4, Supp Fig 1, Supp Fig 2 |
| Synthesis of results | 21 | Present results of each meta-analysis done, including confidence intervals and measures of consistency.  **Overall pooled prevalence of probable PTSD**  Prevalence estimates had reporting time frames of one week to one year, and estimates were highly variable, ranging from 0% (95% CI: 0%-0%) to 74% (95% CI: 72%-76%), and heterogeneous (Q=18,326.70, df=24, p<.001); I^2^ = 99.87%. Overall, pooled prevalence across all studies was 22% (95% CI = 13% - 32%). See Figures 2 and 3. Pooled estimates were recalculated only including the 20 studies that achieved at least an 80% quality score. The pooled estimate from the high quality studies was 20% (95% CI=11% to 32%). There was no association between study quality and prevalence (b=-.001 (95% CI = -.11, .11), p=.98). | Results, Overall pooled prevalence of probable PTSD, 1 and  Figure 3 |
| Risk of bias across studies | 22 | Present results of any assessment of risk of bias across studies (see Item 15).  Third, as noted above, there was high heterogeneity in the prevalence estimates with an I^2^ of more than 99%. We were therefore unable to statistically assess the risk of publication bias in this meta-analysis because none of the publication bias methods provide accurate results with more than moderate levels of heterogeneity (i.e. I^2^<50%) (123). We are therefore unable to provide insight into the level of publication bias that may be present in these results. | Discussion, Limitations, 4 |
| Additional analysis | 23 | Give results of additional analyses, if done (e.g., sensitivity or subgroup analyses, meta-regression [see Item 16]).  There was no association between study quality and prevalence (b=-.001 (95% CI = -.11, .11), p=.98).  **Pooled prevalence by reporting time frame**  The two WHO Mental Health Survey studies (69, 77) were the only ones to use a one-year time frame. Because Ayazi et al (2012)’s (88) study was the only study that utilized a two week time frame, it was grouped with those using a one week time frame when stratifying by assessment time frame, since this study used the HTQ (102), which has a one week assessment time frame (102). Across all the studies, there were significant differences by assessment time frame (Random heterogeneity test between sub-groups = 79.61, df=2, *p*<0.001; see Supplementary Figure 2). However, this difference is explained by the two studies that assessed PTSD over a one-year time frame. These two studies (Gureje et al., 2006 (69)) and Herman et al., 2009 (77)) were part of the cross-national World Mental Health Surveys (111) and were the only two studies to use the CIDI (104). They each had pooled prevalence estimates of 0% (95% CI: 0% to 0%). In contrast, the pooled prevalence in the past week was 22% (95% CI=9% to 38%) and the pooled prevalence in the past month was 27% (95% CI=16% to 40%). There was no significant difference in the weekly vs. monthly pooled prevalence (Random heterogeneity test between sub-groups = 0.28, df=1, *p*<=.60). The overall pooled prevalence of probable current PTSD, defined as a period prevalence ranging from one week to one month was 25% (95% CI: 16% to 36%).  **Pooled prevalence by screener vs. diagnostic assessment tool**  There was not a significant difference in the prevalence estimates between studies that used screening instruments (27%, (95% CI: 15%-41%) compared to those using diagnostic structured and semi-structured interviews (14%, [(95% CI: 4%-29%); Random test for heterogeneity between subgroups = 1.76, df=1, *p*=0.18].  **Pooled prevalence by exposure to war or armed conflict**  There was a significant difference in pooled prevalence estimates between studies that were conducted in regions exposed to mass-casualty war or armed conflict at any point during the lifetime of the participants and those that were unexposed (Random test for heterogeneity between subgroups = 13.64, df=1, *p*=0.01) (see Figure 4). The pooled prevalence estimate of studies from exposed regions was 30% (95% CI: 20%-40%), while the estimate from unexposed regions was 8% (95% CI: 3%-15%). Results of a meta-regression indicated that the adjusted R-square indicated that conflict exposure accounted for 22.95% of the variance in the pooled prevalence estimates. The pooled prevalence of probable current PTSD in regions exposed to war was 30% (95% CI: 21% to 40%) while the pooled prevalence of probable current PTSD in regions unexposed to war was 13% (95% CI: 4% to 26%); Random test for heterogeneity between subgroups = 4.47, df=1, p=.03 (see Supplementary Figure 3).  **Pooled prevalence by gender**  Five studies (66, 70, 75, 76, 92) did not report prevalence estimates by gender and so they were not included in the analysis of prevalence by gender. Herman et al., 2009 (77) and Gureje et al., 2006 (69) did not report prevalence by gender but did report that there was no significant difference by gender, and so the overall mean score was included as the score for both men and women. Machisa et al., 2017 (84) only sampled women, and so the data from this study was included in the pooled analysis for women but not men.  Of the 19 studies that reported data comparing the rates of PTSD in men and women, 12 reported a significant difference (see Table 1). Of the 12 studies that reported a significant difference in the prevalence of PTSD in men compared to women, 10 reported higher rates of PTSD in women and two reported higher rates of PTSD in men. There were no significant differences in the pooled prevalence of PTSD by gender (Test of heterogeneity between groups = 0.42, df=1, *p*=0.52). The pooled prevalence estimate for females was 25% (95% CI = 14% - 39%). The pooled prevalence estimate for males was 20% (95% CI = 10% - 31%) (see Supplementary Figure 1). Post-hoc meta-regression indicated that the lack of a significant difference in probable PTSD prevalence by gender persisted when predictors included conflict exposure, reporting time frame, and assessment tool type (b = -.08 (95% CI = -.19, .04), *p*=.18). | Results,  Overall pooled Prevalence of PTSD, 1 (last line); Figures 3, 4, Supp Fig 1, Supp Fig 2, Supp Fig 3 |
| **DISCUSSION** | | |  |
| Summary of evidence | 24 | Summarize the main findings including the strength of evidence for each main outcome; consider their relevance to key groups (e.g., healthcare providers, users, and policy makers).  The goal of this review was to provide a more complete picture of the population-based data on the current prevalence of PTSD in SSA than is available from any single study. We identified 25 studies assessing 58,887 individuals in the SSA region. There were four key findings in the meta-analysis: (1) The overall pooled prevalence across all studies, including two studies that calculated a one-year prevalence estimate, was 22% (95% CI = 13% - 32%). The pooled prevalence of current symptoms (defined as duration of one week to one month) consistent with a probable diagnosis of PTSD in SSA was 25% (95% CI: 16% to 36%). (2) Prevalence estimates of current symptoms across the individual studies were highly variable, ranging from 2% (95% CI: 2%-2%) to 74% (95% CI: 72%-76%). (3) There was no significant difference in the pooled prevalence of PTSD for men and women, for reporting time frame or for whether the study used a screening versus diagnostic instrument. (4) Prevalence estimates across regions differed substantially by population level exposure to war or armed conflict. We discuss these findings in more detail below.  The high 22% prevalence of probable PTSD found in this meta-analysis seems to be driven in large part by the 30% prevalence of probable PTSD found across the 17 studies that were conducted on populations exposed to war and armed conflict. The finding that populations experiencing war or armed conflict within the lifetime of the study participants have higher probable PTSD prevalence is consistent with expectations. Countries with higher rates of conflict have populations exposed to higher levels of trauma and, thus, more PTSD symptoms. However, the prevalence of PTSD of 30% found in this meta-analysis still exceeds the rates found in three other meta-analyses in populations exposed to war of 15%(50), 26% (112), and 24%(51). Moreover, the 8% prevalence of PTSD in non-war exposed populations exceeds 4% the rate of PTSD prevalence found in other population-based cross-national studies (2), suggesting that there was still a substantial population level burden of PTSD symptoms in non-conflict affected countries, which a burden that has often been overlooked. Taken together, these data suggest PTSD symptoms and probable PTSD are common in SSA. The higher prevalence of PTSD found in this meta-analysis may be partially explained by studies finding that people living in SSA may be disproportionately affected by individual and population level trauma exposure(8-12, 14), and that the vast majority of people in SSA have very low access to mental health treatment (15, 16). These factors may result in an outsized effect on the population burden of PTSD in SSA (22, 23).  There was no consistent pattern in the results for gender differences in PTSD prevalence. While 10 of the 19 studies that reported on gender differences in PTSD prevalence reported that women had higher rates of probable PTSD, seven studies reported no gender difference, and two studies reported that men had higher rates of probable PTSD. However, there was no difference in the overall pooled prevalence by gender. These findings suggest that gender may frequently be a critical variable to consider when understanding PTSD prevalence, but that its explanatory power may vary by population context.  There was not a significant difference in prevalence estimates between studies that used screening instruments compared to those using diagnostic structured and semi-structured interviews. This result is inconsistent with the results of a meta-regression of prevalence of PTSD in countries experiencing war and armed conflict that found that symptom scales produced prevalence estimates that were 1.5 to 2 times higher than diagnostic tools (50). The authors of the meta-regression suggest that this discrepancy may occur because symptom scales do not assess clinical significance or functional impairment, and may therefore overestimate the prevalence of PTSD (50). While the lack of data on functional impairment in symptom scales is certainly problematic, studies have found a strong correlation between elevated distress on symptom scales and functional impairment (113-115). Indeed, subthreshold PTSD is associated with high distress and impairment and increased risk of suicidality globally (116). Given that only one study used an instrument for PTSD that had been validated in the population of interest, we are unable to conclude if symptom scales or diagnostic instruments may be under or overestimating PTSD. | Discussion, 1-6 |
| Limitations | 25 | Discuss limitations at study and outcome level (e.g., risk of bias), and at review-level (e.g., incomplete retrieval of identified research, reporting bias).  **Limitations**  In producing pooled estimates, we encountered at least four limitations of the identified studies that limit our ability to make interpretations from our findings. First, only one study (Ertl et al., 2014 (92, 93)) used a measure of PTSD symptoms whose reliability and validity had been assessed previously in the population of interest. PTSD symptoms are highly prevalent in the SSA countries where they have been studied. However, the lack of reliable and valid instruments used in the populations studied is a major limitation for both research and practice. In a 2018 review of qualitative studies examining post-trauma symptoms across cultural contexts, most study participants reported symptoms consistent with Western diagnostic criteria but also reported a number of symptoms not captured in those diagnostic classifications (117), supporting the need for local contextualization of measures. Measures that are not locally contextualized and valid may result in substantial under- or over- reporting of trauma exposure and PTSD symptoms. For example, a study of Zulu speaking participants in northeastern KwaZulu-Natal, South Africa found that the PTSD section of the Structured Clinical Interview for DSM Disorders, Axis I, Research Version (SCID-I RV)(118) undercounted participants who had been exposed to traumatic events by almost 20% compared to a Zulu Culture-Specific Trauma Experience Questionnaire (119). Pragmatic and feasible approaches to cultural and contextual validation of measures of post-trauma symptoms in challenging settings have been described (120) and need to become more widely employed prior to undertaking population surveys. Rigorous epidemiological research to examine the predictive validity of PTSD constructs in diverse settings is also an important priority.  Second, although data were available from a relatively large number of participants, the studies only represent data from 10 of the 48 SSA countries, and only six studies provided national level data. There is a complete absence of national or regional population-based data on PTSD from more than 80% of SSA countries, including those affected by ongoing fragile and conflict affected situations, such as Central African Republic, Mali, and Somalia. Given the enormous heterogeneity expected not only across the continent, but also within countries and regions, this review cannot speak to rates of PTSD in any of these other regions. Thus, substantial gaps in our knowledge of PTSD prevalence in the majority of SSA remain.  These disparities in PTSD prevalence data parallel the lack of mental health data and services broadly in SSA (1). Despite the enormous burden mental disorders are projected to pose in SSA by 2050, they remain a low priority in terms of policy initiatives and research funding (121). Even with political will and support, many countries may have difficulty meeting identified mental health needs given limited resources and competing priorities. Indeed, although 72% of countries in the Africa region reported that they had a stand-alone mental health policy, only 27% had allocated resources towards implementing that plan (122).  Third, as noted above, there was high heterogeneity in the prevalence estimates with an I^2^ of more than 99%. We were therefore unable to statistically assess the risk of publication bias in this meta-analysis because none of the publication bias methods provide accurate results with more than moderate levels of heterogeneity (i.e. I^2^<50%) (123). We are therefore unable to provide insight into the level of publication bias that may be present in these results.  Finally, the goal of this review was to focus on adult PTSD and we excluded studies that focused exclusively or primarily on participants who are younger than 15 years old, which is the youngest age that the World Bank considers part of the “working age population” and is the youngest age of majority in countries throughout the world. All of the identified studies had participants whose mean age was 18 years old or older. However, five studies (64, 71, 86, 92) included individuals who were younger than 18 years old, and therefore some of the data comes from adolescents. However, when these studies were removed from the analyses, the results were unchanged.  In addition, there are several limitations in the methodology of our review that should be noted. Our inclusion criteria required that studies have at least 450 participants, used a probabilistic sampling procedure, and reported a quantitative estimate of PTSD. As a result, we excluded smaller studies, those that focused on special populations, and those that reported only continuous measures of PTSD symptoms. Our review does not, therefore, speak to subthreshold PTSD, PTSD in specific populations (e.g. refugees, people living with HIV, students, or combatants), or nuances in context that may impact PTSD presentation and prevalence, such as internally displaced people currently living in conflict zones compared to those living in safe environments. | Discussion, Limitations, 1-6 |
| Conclusions | 26 | Provide a general interpretation of the results in the context of other evidence, and implications for future research.  See response to item 24 and the following:  **Disparities between our pooled estimates and WMH surveys**  The pooled prevalence of probable PTSD in this systematic review (computed from one week to one year prevalence rates) is extremely high when compared to the prevalence reported by a 2017 summary of data from the WMH Surveys. For example Karam et al. (2014) (4) reported a WMH 12-month survey prevalence of 1.1% ranging from 3.8% in Northern Ireland to a low of 0.2-0.3 % in Beijing and Shanghai in the People’s Republic of China, 0.3% in Colombia, and 0.3% in Mexico (4). Indeed, the current PTSD pooled prevalence found in this study far exceeds the 3.9% lifetime rate found in the WMH surveys (2). There are at least four possible reasons for the disparity found between the pooled PTSD prevalence estimates identified in this meta-analysis and the prevalence estimates found in the WMH surveys. First, although our review reports data from only 10 countries, six of those countries have experienced war and armed conflict during the lifetime of the participants. Very few such countries are included in the WMH Surveys, with Iraq and Lebanon being notable exceptions (4). However, even in Iraq and Lebanon, the prevalence of PTSD diagnosis is very low compared with the estimates of prevalence estimates observed in SSA. Second, many of the studies in our pooled estimate specifically focused on PTSD assessment as a primary aim of the study. The WMH survey, in contrast, aims to document the population burden of all mental and behavioral disorders globally without one particular focus. In addition, in most cases the survey is administered in two stages, and PTSD is only included in the second stage of the survey (124). Third, there are specific assessment nuances of the CIDI (111) (WMH survey studies were the only ones that used the CIDI) that may have impacted PTSD symptom report. First, in this review, the CIDI was the only diagnostic interview that was lay-administered. Another key issue with the CIDI, as compared to other instruments used in most of the studies, is the skip out related to reporting trauma. That is, PTSD is only assessed in persons who reported a qualifying trauma. Thus, if a participant does not report a trauma, either because their trauma is not on the list queried or because the person chooses not to report such sensitive information, then these participants do not get assessed for PTSD (124). As a result, PTSD symptoms may go undetected. This is particularly problematic when the trauma events have not been culturally and contextually validated (119). There is some data to suggest that the results of trauma may be underreported in the WMH surveys. For example, the lifetime exposure of sexual violence in the South African WMH survey found a prevalence rate of rape of 2%, however, other epidemiological surveys from South Africa have found lifetime rates of rape between 4.5% and 12% (125).  Fourth, there may be differences in the way that individuals from different countries, communities, and cultures respond to items which may result in under or overreporting of symptoms. The CIDI, like many highly structured interviews, applies an algorithm of combinations of types of symptoms following the Diagnostic and Statistical Manual version IV (DSM-IV) (126) in order to produce a diagnosis. The algorithms, like the diagnostic criteria in DSM-IV, are derived predominantly from Western populations, which give weight to the core symptoms that are known to have salience in those settings but not necessarily in other settings. Concerns about the cross-cultural applicability of the CIDI have been reported in Nepal (127), Ethiopia (128), and American Indian Reservation populations (129). Indeed, a study that used latent-class analysis to re-examine the depression results of the WMH surveys from the US, New Zealand, South Africa, and Nigeria found that participants in Nigeria and South Africa who endorsed the screening questions endorsed more severe depression symptoms that participants from the US and New Zealand who endorsed the screening questions (130). When these differential patterns of responding were taken into account, the prevalence estimate for depression in Nigeria was highest (22%), whereas the results of the Nigeria WMH survey found that it had the lowest rate of depression (3%). Studies are needed that examine the reliability and validity of the diagnostic criteria of PTSD as well as widely used assessments such as the CIDI in SSA populations.  **Conclusions and directions for future study**  Methodological limitations of the extant literature as described above lead us to conclude our pooled estimate should be interpreted with caution. Given limited information on the reliability and validity of the assessment tools used and the lack of data available from most countries in the region, more work is needed before strong conclusions can be made about the population burden of PTSD in SSA. Furthermore, the identified studies provide little or no evidence regarding the proportion of the population requiring specific levels of intervention and thus do little to inform service planning.  However, even given these limitations, our findings suggest that health systems in SSA need to improve the identification and access to treatment for persons with PTSD. The percentage of individuals with PTSD seeking treatment is globally low, and most strikingly low in low-lower middle and upper-middle income countries (2). Improving detection of trauma and PTSD in primary care may be an efficient strategy, given that globally people with PTSD are more likely to seek care in general health settings than in specialty mental health clinics (2). However, major efforts to scale up mental health care, such as the PRIME (**PR**ogramme for **I**mproving **M**ental health car**E**) (131), which is focused on improving mental health care in non-humanitarian settings in several low- and middle- income countries through integration with maternal and primary care, have not, to date, included PTSD as a target disorder. This is likely, in part, due to a lack of data on the prevalence of PTSD and the burden it poses in these settings. Moreover, misconceptions about PTSD remain. For example, PTSD is more widely recognized as a problem for refugees, in high conflict situations and in humanitarian crises. PTSD is included in mental health Gap Action Programme (mhGAP) Intervention Guide for humanitarian settings, but not yet included in the primary mhGAP (132, 133). However, epidemiologic data suggest that, globally, sexual violence in the context of intimate partnerships is responsible for the largest burden of PTSD (134). Thus, much foundational epidemiological work remains to be done to document the burden of PTSD in SSA. This work will be critical to inform policy, research, and treatment and, above all, to address issues of access to care and decrease the burden of PTSD in SSA. | Discussion,  1-15 |
| **FUNDING** | | |  |
| Funding | 27 | Describe sources of funding for the systematic review and other support (e.g., supply of data); role of funders for the systematic review.  This study was supported by the National Institute of Mental Health (L.N., #K23MH110601), by Cohen Veterans Bioscience (K.C.K), by the UK Department for International Development (DfID) (C.H., #201446) as part of the Programme for Improving Mental health carE (PRIME), by the National Institute of Health Research (NIHR) Global Health Research Unit on Health System Strengthening in Sub-Saharan Africa, King’s College London (C.H., GHRU 16/136/54), and by AMARI as part of the DELTAS Africa Initiative (C.H., DEL-15-01). The views expressed in this article do not necessarily reflect the UK Government’s official policies, those of the NHS, the NIHR or the Department of Health and Social Care. The funders had no role in study design, data collection and analysis, decision to publish, or preparation of the manuscript. | Submission form |

*From:*  Moher D, Liberati A, Tetzlaff J, Altman DG, The PRISMA Group (2009). Preferred Reporting Items for Systematic Reviews and Meta-Analyses: The PRISMA Statement. PLoS Med 6(7): e1000097. doi:10.1371/journal.pmed1000097

For more information, visit: **www.prisma-statement.org**.

Page 2 of 2
