# Supplementary figures and images for: National and regional prevalence of posttraumatic stress disorder in sub-Saharan Africa: A systematic review and meta-analysis
Source: PLoS Med. 2020 May 15;17(5):e1003090. doi: 10.1371/journal.pmed.1003090 (PMC7228043; doi:10.1371/journal.pmed.1003090)

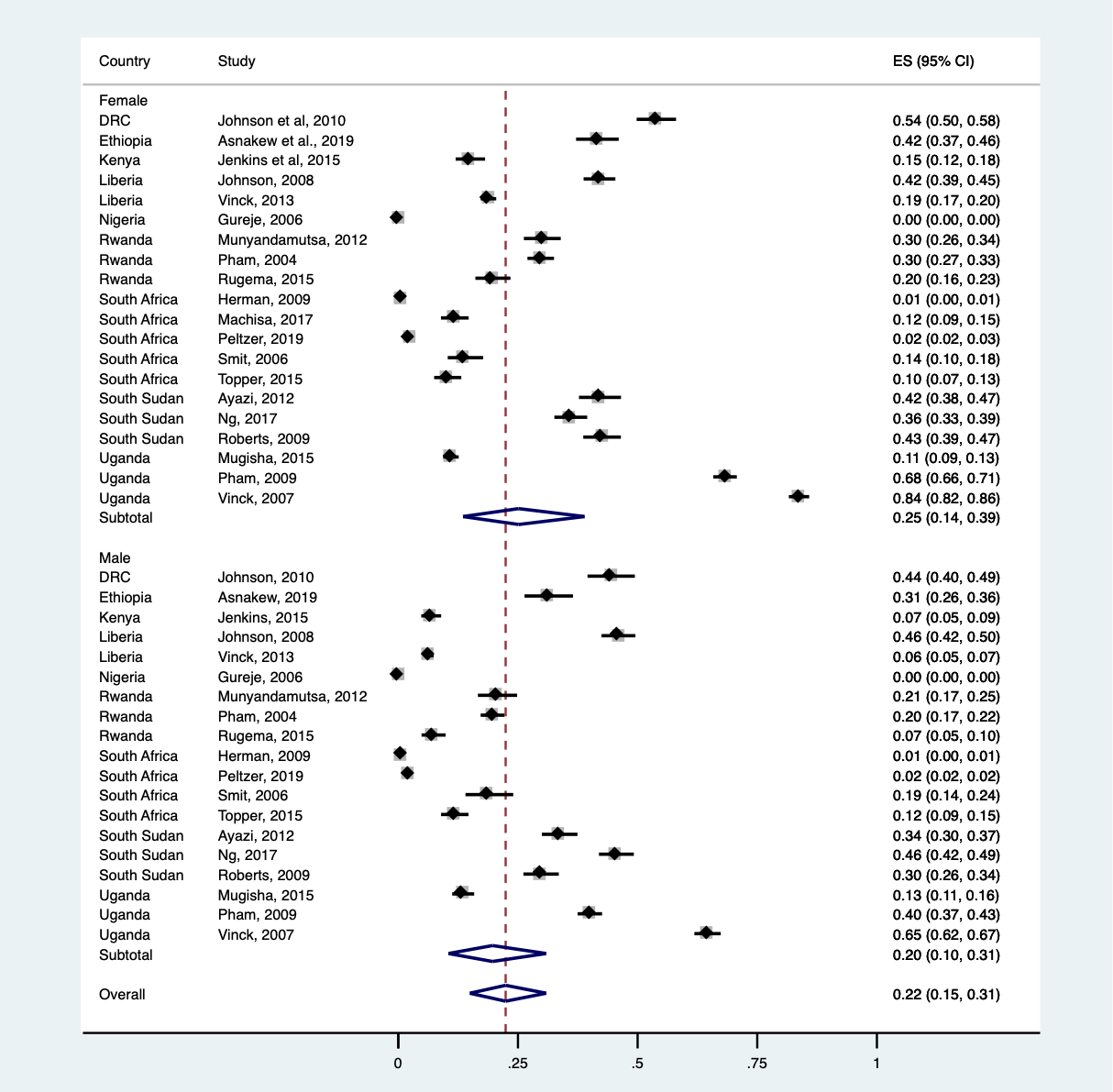

Supplement: S1 Fig — Random test of herterogeneity between subgroups: 0.42, df = 1, p = 0.052. CI, confidence interval; ES, effect size (proportion) (TIFF) [file pmed.1003090.s002.tiff]

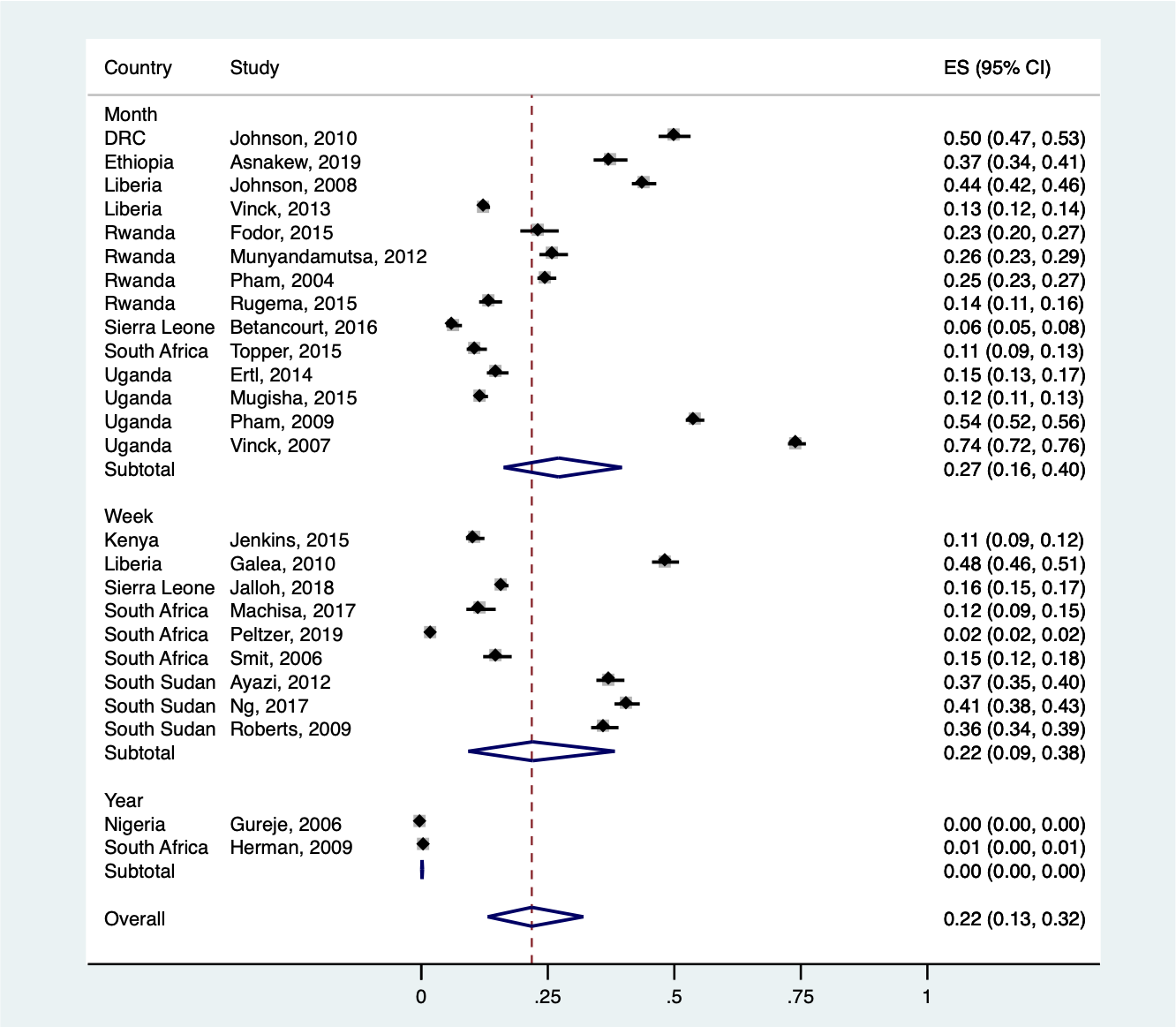

Supplement: S2 Fig — Random test of heterogeneity between subgroups: 79.61, df = 2, p < 0.001. CI, confidence interval; ES = efect size (proportion) (TIFF) [file pmed.1003090.s003.tiff]
